# Four-dimensional measurement of root system development using time-series three-dimensional volumetric data analysis by backward prediction

**DOI:** 10.1186/s13007-022-00968-x

**Published:** 2022-12-09

**Authors:** Shota Teramoto, Yusaku Uga

**Affiliations:** Institute of Crop Sciences, National Agriculture & Food Research Organization, Tsukuba, Ibaraki 305-8602 Japan

**Keywords:** Back prediction, Crown root, Image analysis, Image processing, Nodal root, Radicle, Root growth measurement, Root system architecture, Seminal root, Sequential images

## Abstract

**Background:**

Root system architecture (RSA) is an essential characteristic for efficient water and nutrient absorption in terrestrial plants; its plasticity enables plants to respond to different soil environments. Better understanding of root plasticity is important in developing stress-tolerant crops. Non-invasive techniques that can measure roots in soils nondestructively, such as X-ray computed tomography (CT), are useful to evaluate RSA plasticity. However, although RSA plasticity can be measured by tracking individual root growth, only a few methods are available for tracking individual roots from time-series three-dimensional (3D) images.

**Results:**

We developed a semi-automatic workflow that tracks individual root growth by vectorizing RSA from time-series 3D images via two major steps. The first step involves 3D alignment of the time-series RSA images by iterative closest point registration with point clouds generated by high-intensity particles in potted soils. This alignment ensures that the time-series RSA images overlap. The second step consists of backward prediction of vectorization, which is based on the phenomenon that the root length of the RSA vector at the earlier time point is shorter than that at the last time point. In other words, when CT scanning is performed at time point A and again at time point B for the same pot, the CT data and RSA vectors at time points A and B will almost overlap, but not where the roots have grown. We assumed that given a manually created RSA vector at the last time point of the time series, all RSA vectors except those at the last time point could be automatically predicted by referring to the corresponding RSA images. Using 21 time-series CT volumes of a potted plant of upland rice (*Oryza sativa*), this workflow revealed that the root elongation speed increased with age. Compared with a workflow that does not use backward prediction, the workflow with backward prediction reduced the manual labor time by 95%.

**Conclusions:**

We developed a workflow to efficiently generate time-series RSA vectors from time-series X-ray CT volumes. We named this workflow 'RSAtrace4D' and are confident that it can be applied to the time-series analysis of RSA development and plasticity.

**Supplementary Information:**

The online version contains supplementary material available at 10.1186/s13007-022-00968-x.

## Background

Plant roots are essential for water and nutrient uptake from the soil. Root growth pattern through the soil volume is a major determinant of the ability of plants to absorb water and nutrients [1]. For example, by growing roots in the soil area with fertilizer or water, roots can absorb water and/or nutrients efficiently [2–5]. Conversely, by avoiding root growth in polluted soils, roots do not absorb substances detrimental to plant growth [6]. The resulting specific root growth pattern is known as root system architecture (RSA) [1] and this RSA plasticity is a promising breeding target for making crops resilient to stressful soils [7]. However, there are several issues to be addressed when measuring RSA plasticity in soils.

Measurements of RSA plasticity in soils must be nondestructive, because destructive measurements disrupt the three-dimensional (3D) growth pattern of the roots in the soil and make it impossible to observe changes in RSA over time. For example, shovelomics [8], which uses shovels to excavate the roots from the soil to measure RSA traits, or the monolith method [9], which uses boxes or cylinders with an open bottom to collect soil blocks by driving the monolith into the ground, are simple and commonly used destructive methods for RSA analysis; however, neither of them meets the conditions for measuring RSA plasticity. On the other hand, widely used nondestructive measurement techniques, such as the rhizotron, the minirhizotron, or the root box, involve installing transparent plexiglass plates or tubes in the soil to capture images of the roots growing adjacent to the plexiglass surface [10–14]. These nondestructive methods can be used to evaluate RSA development over time by acquiring images of the glass surface and isolating root segments in the images using an image processing approach [15–17]. However, these nondestructive methods are limited to the observation of RSA development occurring on the plexiglass surface, and the installation of plexiglass affects root growth.

Other nondestructive techniques for in vivo root measurement, use 3D imaging with X-ray computed tomography (CT) and magnetic resonance imaging (MRI) to scan the soil profile and create 3D images that include root segments [18]. In most cases, such root segments are extracted using image processing approaches to measure root distribution in the soil as an RSA trait [19–21]. Thus, X-ray CT and MRI effectively overcome the shortcomings mentioned above. However, most studies have only analyzed overall parameters, such as root distribution in the soil but not specific parameters, such as individual root growth rates, as it is difficult to track all individual roots in 3D images. Nonetheless, specific parameters are most important for estimating how each root responds to the particular soil environment in which it is trying to grow, and which varies greatly even over very short distances.Numerous studies have measured the specific parameters of individual roots in two-dimensional images. Thus, for example, in an experiment using a root box with soil substrates, the root elongation rate was measured by tracking rice (*Oryza sativa*) and sorghum (*Sorghum bicolor*) root tips [22]. Similarly, in Arabidopsis (*Arabidopsis thaliana*), the root elongation rate was measured using solid medium plates [23–25]. In both cases, cameras were placed at the root tips to measure root elongation. However, using these methods, only a limited number of roots could be observed, whereby, elongation of the entire RSA could not be measured. Furthermore, even without limiting the dimension, most studies that analyze the entire RSA focus on root distribution [26–28], as the root segmentation and skeletonization required for root distribution measurements are relatively easy. However, the problem is more complicated when a large number of roots is to be tracked. Currently, there are very few methods for measuring specific parameters of all the roots that make up the RSA.

Once roots have extended into different portions of the soil profile, their spatial position remains unchanged unless pressure is applied to the soil or soil volume changes owing to changes in soil moisture content [29, 30]. In other words, provided a root does not change its spatial coordinates once it has elongated, then roots developing at different times will necessarily overlap among themselves, such that matching underground sections at different plant growth stages is relatively easy [27]. In this case, RSA development can be easily calculated from the overlapping sections of the soil using the RSA vector that represents the RSA skeleton as a set of sequences of coordinate points [31, 32]. Therefore, if the RSA vector for all sampling time points is available, we can measure RSA development from the overlapping portions [33]. Nonetheless, the issue with this methodology is how to efficiently generate an RSA vector for all sampling time points. A possible solution is to predict a vector at one time point, given a vector at another time point.

There are two types of predictions: forward and backward. Forward prediction predicts the future after a certain time point, whereas backward prediction predicts the past [34]. Both types of prediction have been used with sequential digital data, such as satellite images at different time points [35] and video frames [34, 36]. We hypothesized that these predictions may be possible for sequential vector data as well. Focusing on the root tip, the forward prediction needs to estimate the direction of root growth, whereas the backward prediction does not need to estimate the direction, because the one-time RSA vector perfectly overlaps with the RSA vector at a previous time point. Therefore, backward prediction is suitable for predicting time-series RSA vectors.

In this study, we developed a workflow that semi-automatically created time-series RSA vectors from X-ray CT images captured every day and calculated the time-series local RSA parameters. This was achieved by using a soil substrate whose volume barely changes with water content [19] and backward prediction to compute the RSA vector at all time points in the time series based on the RSA vector at the last time point of the time series.

## Results

### Workflow without backward prediction

**Fig. 1:**
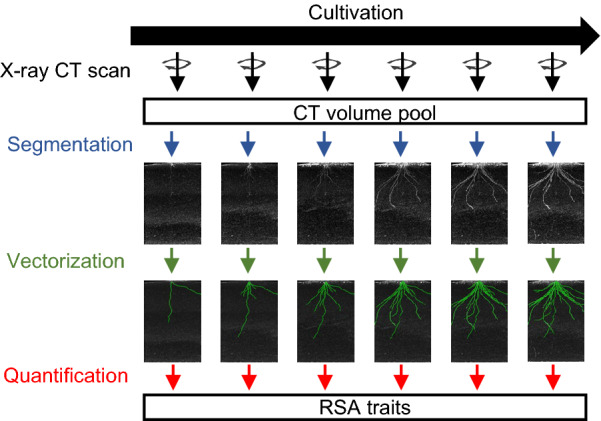
Workflow without backward prediction. Segmentation: CT volumes were converted to RSA-segmented volumes by RSAvis3D. Projection views of 3D RSA-segmented volumes are shown. Vectorization: RSA-segmented volumes were vectorized to RSA vector. Projection views of 3D RSA-segmented volumes merged with 3D vectors. RSA vector is drawn in green

When backward prediction is not used, an RSA vector must be created for all the time points. Figure 1 shows a schematic for measuring RSA traits from time-series X-ray CT volumes without backward prediction. In this example, six X-ray CT scans of a single potted rice plant resulted in six X-ray CT volumes. Each X-ray CT volume was converted to an RSA-segmented volume using the RSA visualization software RSAvis3D [19], and then to the RSA vector using the RSA vectorization software RSAtrace3D [31]. Then, RSA traits were calculated based on the six RSA vectors. Segmentation is fully automated; however, vectorization requires human intervention [19, 31]. Given $n$ number of time points, the effort required is $n$ times greater than when the number of time points is one.

### Workflow with backward prediction

**Fig. 2:**
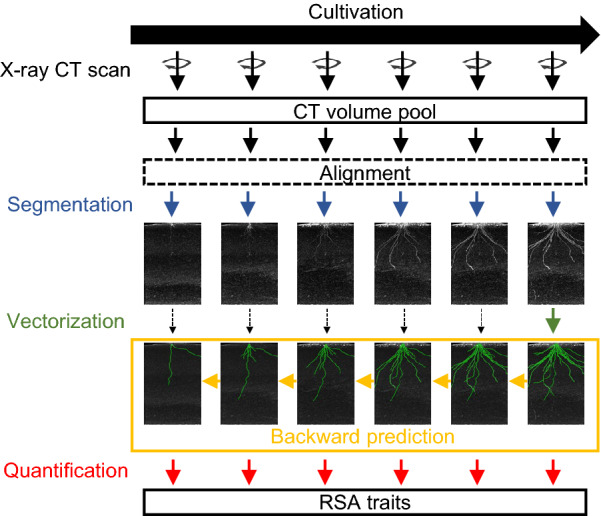
Workflow with backward prediction. Segmentation: CT volumes were converted to RSA-segmented volumes by RSAvis3D. Projection views of 3D RSA-segmented volumes are shown. Vectorization: RSA-segmented volumes were vectorized to an RSA vector. Projection views of 3D RSA-segmented volumes merged with 3D vectors. RSA vector is drawn in green. Backward prediction: The ochre arrows indicate the direction of the backward prediction

Figure 2 shows a schematic diagram for measuring RSA traits from time-series X-ray CT volumes using backward prediction. To perform backward prediction, the time-series CT volumes should be aligned in 3D because the position and angle of the pot on the turntable in the X-ray CT machine are different every day. After alignment, each X-ray CT volume was converted to an RSA-segmented volume. The vectorization step differs from that without backward prediction. We must create an RSA vector only at the last time point of the time series. The remaining RSA vectors at other time points were automatically created by backward prediction. Compared with the workflow without backward prediction, this workflow reduces the amount of work requiring human intervention to $1/n$.

### Volume alignment by registration

**Fig. 3:**
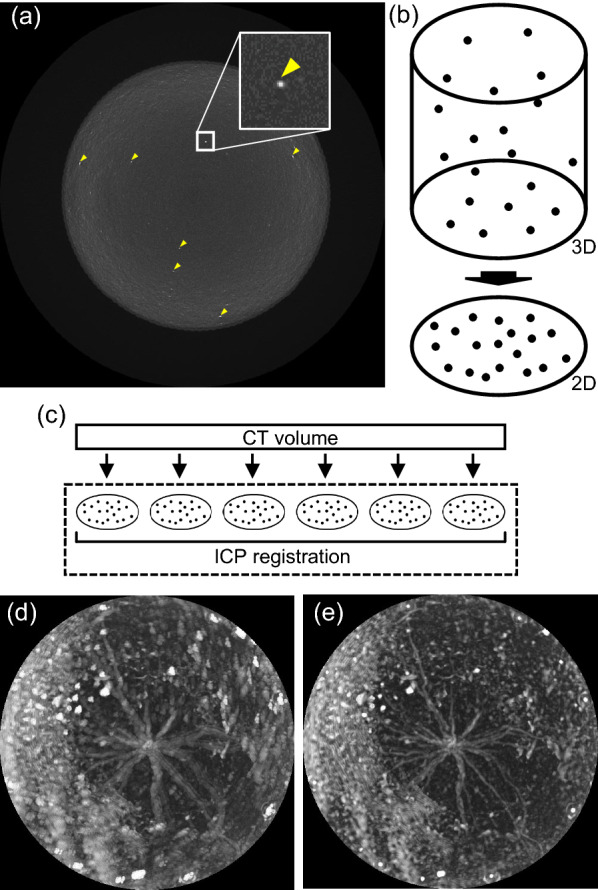
SBI-ICP registration. **a** A x–y slice of an X-ray CT image. Image size is 307.2 mm × 307.2 mm. The yellow arrowhead indicates high intensity signals derived from minerals. **b** A point cloud based on high intensity signals derived from minerals. The point cloud was designated as SBI (soil block identifier). **c** ICP (iterative closest point) registration with time-series X-ray CT volumes. **d** Overlaid top-view projections of 21 RSA-segmented volumes without and **e** with ICP registration

Generally, 3D volume alignment is performed by extracting feature points from a 3D volume and performing registration with these points [37]. However, the feature points of the roots change as roots grow. Therefore, extraction of feature points from the soil substrate is desirable. To this purpose, we focused on particles such as minerals, which show high-intensity signals in the CT images of soil substrates. Minerals absorb X-rays well; therefore, they are represented as white dots in the CT volume (Fig. 3a). A 3D point cloud from the dots in the CT volume was created and converted to a 2D point cloud because the vertical positions did not need to be aligned (Fig. 3b). These point clouds were designated as SBI (Soil Block Identifier). If a soil substrate with minimal volume changes is used, the time-series SBI should be identical. We performed ICP (iterative closest point) registration [38] with the time-series SBI to align the time-series CT volumes (Fig. 3c). ICP registration using SBI was designated SBI-ICP registration. Subsequently, we used 27 CT volumes of rice RSA from 7 to 27 days after sowing (DAS) as sample materials (Teramoto et al., 2020). Figure 3d, e, show the overlaid images of 21 top-view projections of rice RSA from 7 to 27 DAS with and without SBI-ICP registration, respectively. Without SBI-ICP registration, the overlaid image was blurred (Fig. 3d), whereas, it became clear with SBI-ICP registration (Fig. 3e). The sequential animations are shown in Additional file 1: Movie S1. These results indicate that the SBI-ICP registration fully aligned CT volumes.

### Overview of backward prediction

**Fig. 4:**
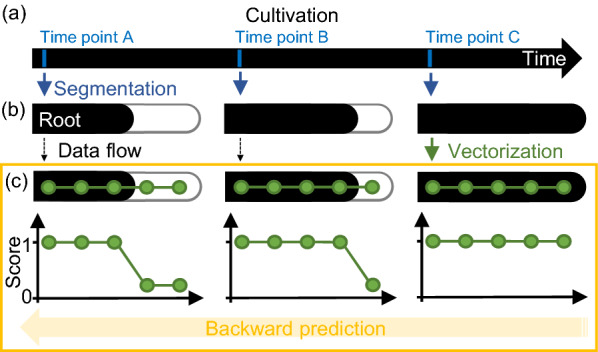
Overview of backward prediction in this study. **a** Time course diagram of cultivation. There are three sampling time points, A, B, and C. The last data of the time series is obtained at sampling time point C. **b** Schematic diagram showing that the roots are shorter before a certain time point. The black segment indicates a root at time points A, B, and C. **c** Schematic diagram of overlaying a root segment drawn in (**b**) and a RSA vector. Green circles indicate vector nodes and green lines indicate a connection between the nodes. Each node was scored based on whether the node overlapped with the root segment. RSA vector at sampling time point C should be manually prepared. RSA vectors at sampling time points A and B are automatically predicted by backward prediction

A schematic of backward prediction is shown in Fig. 4. Given three root segments at three different time points (Fig. 4a), the root segments at earlier time points should be shorter than the root segment at the last time point (Fig. 4b). If we can determine how much shorter it is, backward prediction is possible. The RSA vector at the last time point will be overlaid completely on the root segment at the last time point, but incompletely on the root segment at the earlier time point (Fig. 4c). Therefore, with a discriminator that can judge whether the vector node overlaps with the root segment or not, we can estimate the length of the root segment and perform backward prediction.

### Discriminator for overlapping

**Fig. 5:**
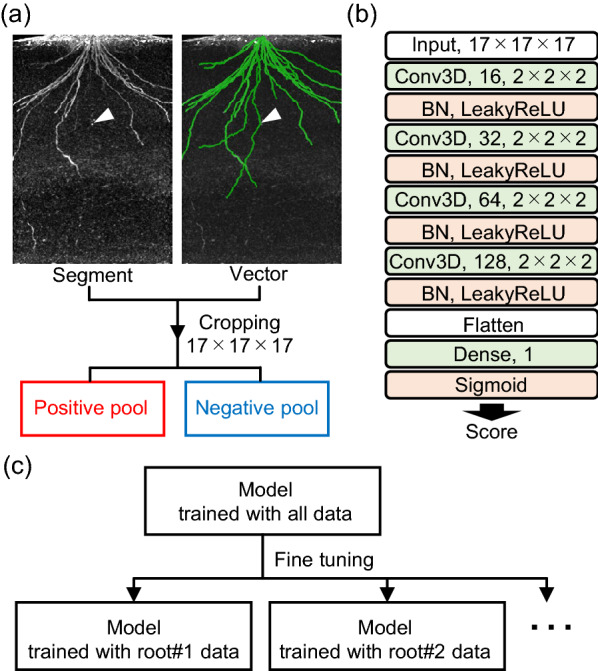
Discriminator for overlapping. **a** Based on the vector node coordinates, blocks with size 17 × 17 × 17 voxels were cropped as positive and negative training data. The white arrowheads indicate faint root segments. **b** Convolutional neural network of the discriminator. Conv3D: 3D convolution, BN, batch normalization. **c** Fine-tuning of the discriminator model for each root

We designed a convolutional neural network (CNN)-based simple discriminator to determine the overlap between the vector node and the root segment. This discriminator was trained using the RSA segments and vector data at the last time point. Volume blocks (17 × 17 × 17) were sampled from the RSA-segmented data as positive and negative training data, and positive and negative data were collected at coordinates where vector nodes were located and not located, respectively (Fig. 5a). The blocks were converted to scalars using the CNN, and the scalars were normalized to 0–1 using the sigmoid function as scores (Fig. 5b). A score of 1 indicated that the input data was an RSA segment and a score of 0 indicated that it was not. Because scoring of faint segments, such as a seminal root (indicated by arrowheads in Fig. 5a), by the discriminator trained with all vector data was difficult, sub-discriminators fine-tuned by roots were used for backward prediction (Fig. 5c).

### Fully automated backward prediction

**Fig. 6:**
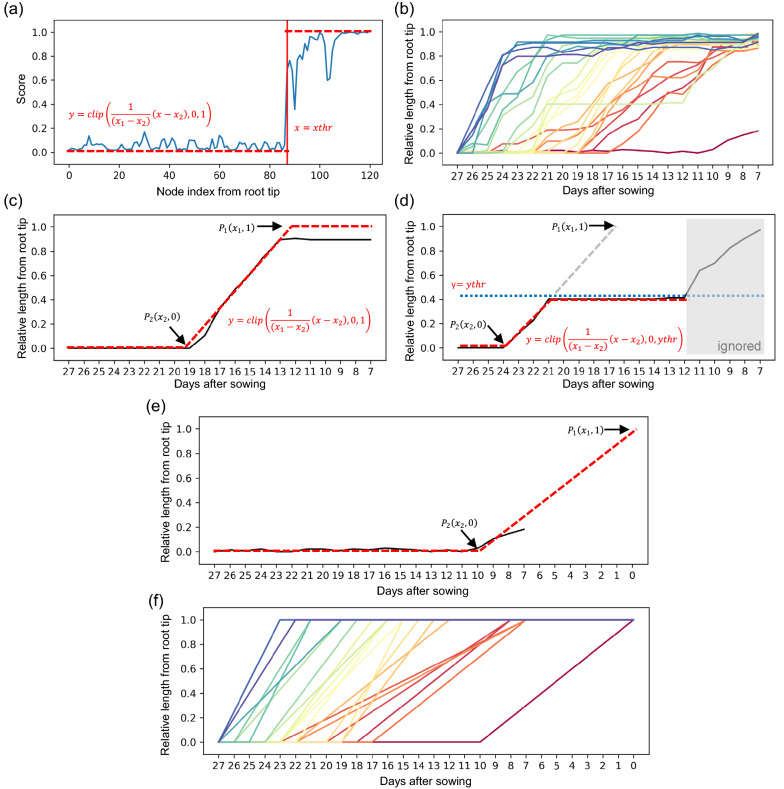
A test case of backward prediction. Time-series X-ray CT volumes from 7 to 27 days after sowing was used. **a** An example of backward prediction of a root. Node indexes (the number of nodes counted from the root tip) and scores calculated by the discriminator were plotted. The red dashed line is represented by the equation in the figure. **b** An example of backward prediction of all 25 roots. Node index was converted into relative length (RL) from the root tip; 0.0 indicates the full-length and 1.0 indicates the root with length 0. Each trajectory corresponded to each root. **c** An example of fitting for basic trajectories. A trajectory was approximated with a line having two bending points. The red dashed line is represented by the equation in the figure. **d** An example of fitting for overlapped trajectories. **e** An example of fitting for incomplete trajectories. **f** All 25 trajectories after approximation

We vectorized the RSA-segmented volume of the last day (27 DAS) using RSAtrace3D [31] and performed a backward prediction. An example of backward prediction for one root at 10 DAS is shown in Fig. 6a. Approximately 120 vector nodes were used to represent the roots. The score for each node was calculated using a discriminator, and then plotted. We found a border between scores 0 and 1, indicating that the root length was shortened to the border. For all roots, all borders from 7 to 27 DAS were estimated to calculate the relative length (RL) from root tip (Fig. 6b); 0.0 indicated the full-length and 1.0 indicated roots with length 0. There were 26 roots, but one root (Root #02) did not show a change in RL (Additional file 2: Movie S2) because this root was full-length at 7 DAS. Thus, 25 trajectories were obtained. The RL of all roots was 0.0 at 27 DAS because the roots at 27 DAS were the longest. Through backward tracing, the RL of each root approached 1.0. Most of the trajectories were represented as lines bent at two points. An example of this is shown in Fig. 6c. The slope of the line indicates that RL changed. In other words, the roots elongated by spending the days it took RL to change from 0.0 to 1.0. The two points of bending can be regarded as the beginning and termination of root elongation. Given that the days of the beginning and ending of root elongation are ${x}_{1}$ and ${x}_{2}$, respectively, and that the starting point is ${P}_{1}\left({x}_{1}, 1\right)$ while the end point is ${P}_{2}\left({x}_{2}, 0\right)$, the two points can be calculated by approximating the trajectory using Eq. (1):

1 $$\begin{array}{c}y=min\left\{max\left[\frac{1}{\left({x}_{1}-{x}_{2}\right)}\left(x-{x}_{2}\right), 0\right],1\right\}\end{array}$$

However, some trajectories, such as those shown in Fig. 6d and e, could not be represented as lines that bent at two points. As shown in Fig. 6d, RL stopped changing approximately at 21 DAS, and approached 0.0 again at approximately 12 DAS. This type of trajectory was created by overlapping with another trajectory from 7 to 21 DAS. Therefore, the slope from 21 to 24 DAS reflected the original root growth. When the period from 7 to 12 DAS was ignored, the trajectory in Fig. 6d could be regarded as a line that bent into two points. Given that $ythr$ is RL when the change in RL stopped, the two points can be calculated by approximating the trajectory using Eq. (2):

2 $$\begin{array}{c}y=min\left\{max\left[\frac{1}{\left({x}_{1}-{x}_{2}\right)}\left(x-{x}_{2}\right), 0\right],ythr\right\}\end{array}$$

As shown in Fig. 6e, RL had already started at 7 DAS, and the slope was interrupted before it reached 1.0. In this case, assuming the slope extends to 0 DAS, the two points can be calculated by approximating the trajectory using Eq. (1). All trajectories after approximation are plotted in Fig. 6f, which represents the elongation pattern of each root.

### Reconstruction of RSA vector and quantification of root growth

**Fig. 7:**
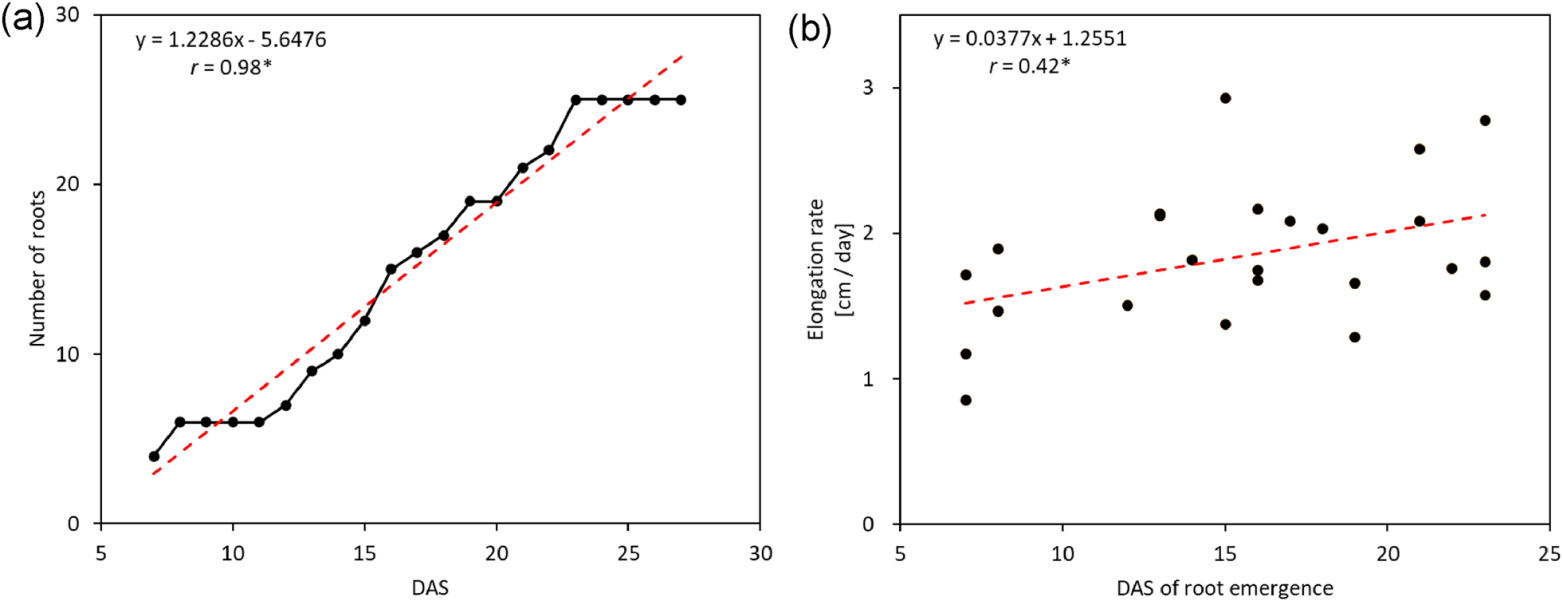
Calculated root growth parameters in rice. **a** Changes in the root number over time. **b** Relationship between days after sowing (DAS) when roots emerge, and root elongation rate. * indicates significant Pearson’s correlation (significance level is 5%)

Using backward-prediction results, RSA vectors at all time points were reconstructed (Additional file 3: Movie S3). This was achieved by shortening the vector at 27 DAS using RL. Vectors almost identical to those created without backward prediction were created. This time-series of vector data was used to evaluate the transition of root number and elongation rate. The root number increased linearly (Fig. 7a), and the elongation rate was significantly higher in late emerging roots (Fig. 7b). These results suggest that the elongation rate of the roots depends on the timing of root emergence.

### Improving efficiency through backward prediction

We evaluated the amount of effort that could be saved by using backward prediction (Table 1). The program was run in a 64-bit Ubuntu 20.04 LTS (CPU: Intel^®^ Core™ i7-8700 CPU@3.20 GHz, memory:32 GB, GPU: NVIDIA GeForce RTX 2080 Ti). Without backward prediction, RSA segmentation and vectorization required 231 min. Instead, with backward prediction, the segmentation time was the same but the vectorization time, which is the only process that requires human intervention, was reduced to 1/21. It takes some additional time for registration and backward prediction, but all the analyses were concluded in 46 min.

**Table 1:** Time required to quantify time-series X-ray CT images for 21 days.

	Manual	Backward prediction
Registration	–	5
Segmentation	21	21
Vectorization	**210**	**10**
Backward prediction	–	10
Quantification	0	0
Total	231	46

Units are minutes. Bold font indicates a process that requires human intervention

## Discussion

An efficient method for creating RSA vectors from time-series 3D volumetric data is required for the measurement of RSA plasticity. In this study, we developed a semi-automatic workflow for this purpose, which consists of SBI-ICP registration based on the distribution of strong signals in soils and backward prediction that automatically creates an RSA vector before a given reference point. Using the time-series RSA vector created, we successfully measured RSA development. Thus, for example, we measured the elongation rate of individual roots. We propose that this workflow is applicable to the study of RSA plasticity. To the best of our knowledge, this is the first study to quantify RSA development by backward prediction of RSA vectors with time-series 3D volumetric data. The implementation of this workflow, which is specified for rice, was named RSAtrace4D and is available at the GitHub repository (https://github.com/st707311g/RSAtrace4D).

RSAtrace4D enables a previously difficult analysis of the growth of the individual roots that make up RSA. Using 21 CT images of an upland rice variety at 21 time points, we demonstrated that the elongation rate of roots was higher at later developmental stages (Fig. 7). If such data can be easily obtained, it can be used not only to evaluate varietal differences but also to evaluate RSA plasticity in response to soil environment. For example, RSAtrace4D could be used for developmental analysis of RSA in response to soil fertility level [26, 39–42], local soil compaction conditions [43–45], or global stress factors, such as drought [46], or high temperature [47]. Therefore, we believe that RSAtrace4D is an effective tool for elucidating the mechanisms of root development in heterogeneous environments and during environmental stress.

Using 21 CT images, we manually vectorized RSA at the last time point of the time series, and the RSA vectors at all time points were automatically created using backward prediction. Given $n$ number of time points, the required labor was calculated as $1/n$, indicating that the greater the number of time-series data, the more labor-saving it is. Additional processing time is required, as noted in Table 1; however, these processes are fully automated and have no impact on labor. The factors contributing to the success of this method are the fixation of roots in soils once they have grown, and alignment of CT volumes with strong signal in the soil (SBI-ICP registration). We used calcined clay Profile^®^ Greens Grade™ (PROFILE Products, Buffalo, Illinois, USA) as a soil-like substrate [19]. Regardless of soil moisture conditions, its features showed little change in volume. Furthermore, the strong signal particles were moderately contaminated (Fig. 3a), making them easy to use for SBI-ICP registration. However, particularly near the base, roots are dense, and there is a risk that the position of high-signal particles near the base may shift as the roots grow. Because ICP registration uses a point cloud and not a single point, some shifting with growth occurred which was not a problem for the month-long cultivation in this study. If SBI-ICP registration is performed using natural soil, the point cloud may be altered due to changes in soil volume during cultivation, and SBI-ICP registration may not work optimally. In such cases, the field soil should be adjusted to mix with a soil substrate whose volume does not change, such as calcined clay, so that the point cloud is not altered. Alternatively, plants should be grown in an environment where the soil volume is not altered.

The method described herein that uses backward prediction has the potential for application to developmental analysis for aboveground parts of RSA, although there are some obstacles. In the current time-series analysis of aboveground traits, organ-level development can be tracked over time by plant part matching at different growth stages [48, 49]. This was achieved by matching the plant parts in 3D mesh data by aligning the 3D mesh data and regarding the overlapping parts as the same parts [49], or by matching the plant parts in 3D point-cloud data by isolating key points encoding both semantic and topology of different growth stages and performing registration of each point [48]. In both cases, there are certain requirements for time-series analysis. The first is to acquire time-series data at a high frequency to facilitate matching. A long interval between successive data acquisition events will result in the inability to perform matching due to changes in plant structure as the plant grows. Second, the matching feature points must be designated. Changing the feature points during growth makes matching more difficult. Therefore, we assumed that backward prediction for the aboveground parts would be possible if scanning intervals were as short as possible, and the shift of feature points should be compensated.

For multiple plants in one volume, we assumed that this workflow would work properly although it is necessary to vectorize each individual plant. Therefore, it is expected to be used to study plant-plant interactions. Furthermore, this workflow is considered applicable even if the type of data changes because the discriminator is retrained for each pot. The registration algorithm may need to be modified when the soil type or other parameters are changed, but the source code is available on GitHub; therefore, researchers are free to modify it. The only step that requires human intervention is the creation of vector data at the final sampling time point. At present, full automation of RSA vectorization is one of the issues that should be solved to accelerate RSA research [50]. Therefore, automatically creating RSA vectors at the last time point is a challenge that needs to be solved to enable fully automatic RSA measurements of time-series image data. Because analysis over time is required for both above- and below-ground structures, such analysis will become more widespread based on this method.

## Conclusions

We developed a semi-automatic workflow for the measurement of 3D root system development from time-series X-ray computed tomography volumes using backward prediction. We observed changes in root elongation rate with growth, which indicated that this workflow is a useful tool for root growth measurement, and that it can be applied to the study of RSA plasticity responses to varying soil environments.

## Methods

We used X-ray CT data as previously described, together which details of the plant materials, growth conditions, and X-ray CT scanning conditions [19]. A brief description of the process is provided below.

### Plant materials and growth conditions

The upland rice variety ‘Kinandang Patong’ (IRGC #23,364) was used in the experiments described herein. Plants were cultivated in a growth chamber for 28 days in 25 cm deep and 20 cm diameter pots (TSP2530P, Tecs, Itako, Ibaraki, Japan) filled with Profile^®^ (Greens Grade™, PROFILE Products, Buffalo, Illinois, USA), an inorganic soil amendment commonly used in cultivation systems for X-ray CT scanning. A hydroponic solution (pH 5.5) consisting of 1.23 mM NO_3_^−^, 0.41 mM NH_4_^+^, 0.18 mM H_2_PO_4_^−^, 1.00 mM SO_4_^2−^, 1.78 mM K^+^, 0.55 mM Mg^2+^, 0.37 mM Ca^2+^, and 8.9 μM Fe^3+^ was used to saturate the growth media and ion-exchanged water was supplied from the pot bottom during cultivation. The growth conditions were 14 h photoperiod regime, temperature from 25 to 30 °C, and humidity from 50 to 60%.

### X-ray CT scanning and root segmentation

Rice roots were imaged daily from 7 to 21 DAS using the X-ray CT system inspeXio SMX-225CT FPD HR (Shimadzu Corporation, Nakagyo-ku, Kyoto, Japan). The scanning conditions were as follows: tube voltage was set at 225 kV, tube current at 500 μA, 1.0-mm Cu (copper) filter, 1200 projections, using a signal averaging two frames over 360° without binning (pixel detector resolution: 3000 × 3000), at 4.0 fps. The final spatial resolution was 0.3 mm, corresponding to a total volume of 30.72 × 30.72 × 25.8 cm^3^. Root segments were isolated using the segmentation software RSAvis3D [19]. To eliminate the roots that changed their growth direction by touching the pot wall, an area 18 cm in diameter was isolated.

### SBI-ICP registration

Python version 3.8.12 was used for SBI-ICP registration [51]. The CT volumes were loaded as NumPy arrays [52]. The regions with high intensity were isolated by thresholding, and their centroids were calculated by the ‘regionprops’ function from scikit-image package version 0.18.3 [53]. The centroids were converted into a 3D point cloud by Open3D package [54] and ICP registration was performed by the ‘registration_icp’ function in Open3D package (0.13.0).

### Backward prediction

Again, Python version 3.8.12 was used for this purpose. The registered CT volumes were converted into RSA-segmented volumes using RSAvis3D. Twenty six roots in the RSA-segmented volume at 27 DAS were vectorized using RSAtrace3D. The 2835 node points that make up the vector were extracted as positive nodes. Negative nodes of the same number were randomly selected such as not to overlap with positive nodes (Fig. 5a). A 17 × 17 × 17 block was extracted from each node. Using 5670 nodes, the U-Net model was trained for 100 epochs. The structure of the model is shown in Fig. 5b. The loss was defined as the mean squared error (MSE), and the Adam optimizer was used at a learning rate of 1e-5. Training was stopped when the loss fell below 0.05 five times consecutively. By fine-tuning the trained model, the submodel for each root was trained again (Fig. 5c).

Using the trained models, the RSA vectors from 7 to 26 DAS were predicted. A score was calculated for each node, and the length of the vector was determined. Then, a graph was created with the x-axis as the node index (the number of nodes counted from the root tip) and the y-axis as the score (Fig. 6a). By approximating this plot with Eq. (3), the borderline between scores of 0 and 1, $xthr$, was calculated.

3 $$ y = f\left( x \right) = \left\{ {\begin{array}{*{20}l}    {0,} \hfill & {x;xthr} \hfill  \\    {1,} \hfill & {x \ge xthr} \hfill  \\   \end{array} } \right. $$

Approximation was performed using brute force, and $xthr$, whose MSE was the smallest, was selected. Thus, $xthr$ was converted into RL, and a graph was created with the x-axis as DAS and the y-axis as RL (Fig. 6b). DAS at the time root elongation began (${x}_{1}$), and when it was concluded (${x}_{2}$), were calculated by approximating each line on the graph with Eq. (4).

4 $$\begin{array}{c}y=f\left(x\right)=min\left\{max\left[\frac{1}{\left({x}_{1}-{x}_{2}\right)}\left(x-{x}_{2}\right), 0\right], ythr\right\}\end{array}$$

where $ythr$ is the y value at which changes in RL are stalled (Fig. 6d). Approximation was performed using brute force, and ${x}_{1}$ and ${x}_{2}$, for which MSE was smallest.

### Calculation of root number and root elongation rate

Root length was calculated from the root length at 27 DAS and RL. Given that the root length on the last day is $Length$ and the root length at *n* DAS is ${Length}_{n}$, this value was calculated using Eq. (5).

5 $$\begin{array}{c}{Length}_{n}=Length\times \left(1-\mathrm{RL}\right)\end{array}$$

If ${Length}_{n}$ is zero or RL is one, the root has not yet initiated at $n$ DAS. The number of roots for which ${Length}_{n}$ was greater than zero was defined as the root number at $n$ DAS. Pearson's correlation test was performed using the ‘cor.test’ function of R, version 3.6.3.

## Supplementary Information

**Additional file 1: Movie S1. **Animations of 21 top-view projections of rice RSA from 7 to 27 DAS with and without SBI-ICP registration.

**Additional file 2: Movie S2. **Animations of backward prediction results from 7 to 27 DAS.

**Additional file 3: Movie S3. **Animations of 21 top-view projections of rice RSA computed using RSA vectors generated by backward prediction. The color scale indicates the depth.

## Data Availability

The datasets used in this study are available at the GitHub repository (https://github.com/st707311g/) and the project homepage (https://rootomics.dna.affrc.go.jp/en/). If not, they are available from the corresponding author on reasonable request.

## Abbreviations

3D: Three-dimensional
CNN: Convolutional neural network
CT: Computed tomography
DAS: Days after sowing
ICP: Iterative closest point
MRI: Magnetic resonance imaging
RL: Relative length
RSA: Root system architecture
SBI: Soil block identifier
