# Supplementary figures and images for: Four-dimensional measurement of root system development using time-series three-dimensional volumetric data analysis by backward prediction
Source: Plant Methods. 2022 Dec 9;18:133. doi: 10.1186/s13007-022-00968-x (PMC9733169; doi:10.1186/s13007-022-00968-x)

## Slide 1
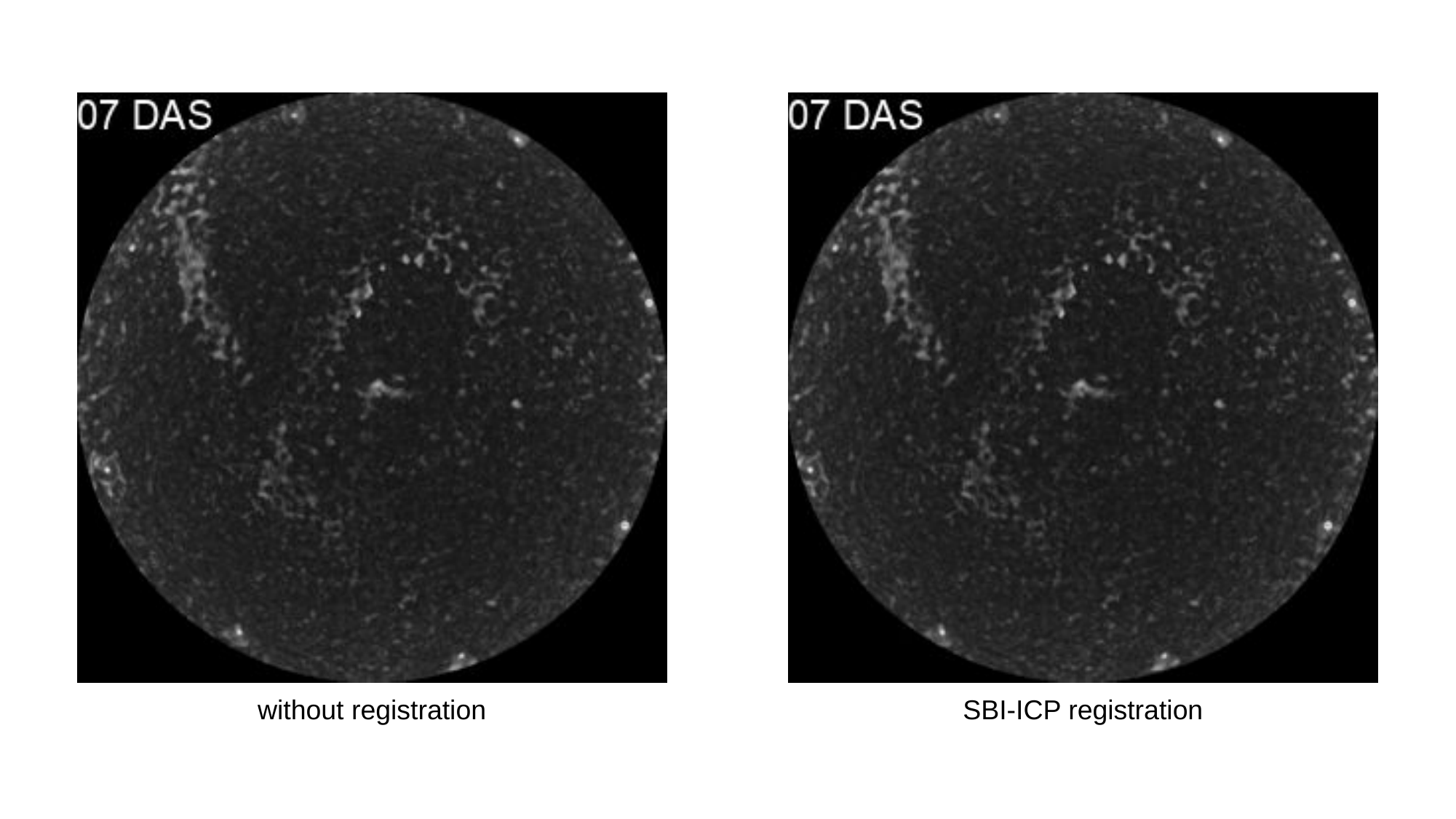

without registration
SBI-ICP registration

Supplement: Supplementary file 1 — Additional file 1: Movie S1. Animations of 21 top-view projections of rice RSA from 7 to 27 DAS with and without SBI-ICP registration. [file 13007_2022_968_MOESM1_ESM.pptx]

## Slide 1
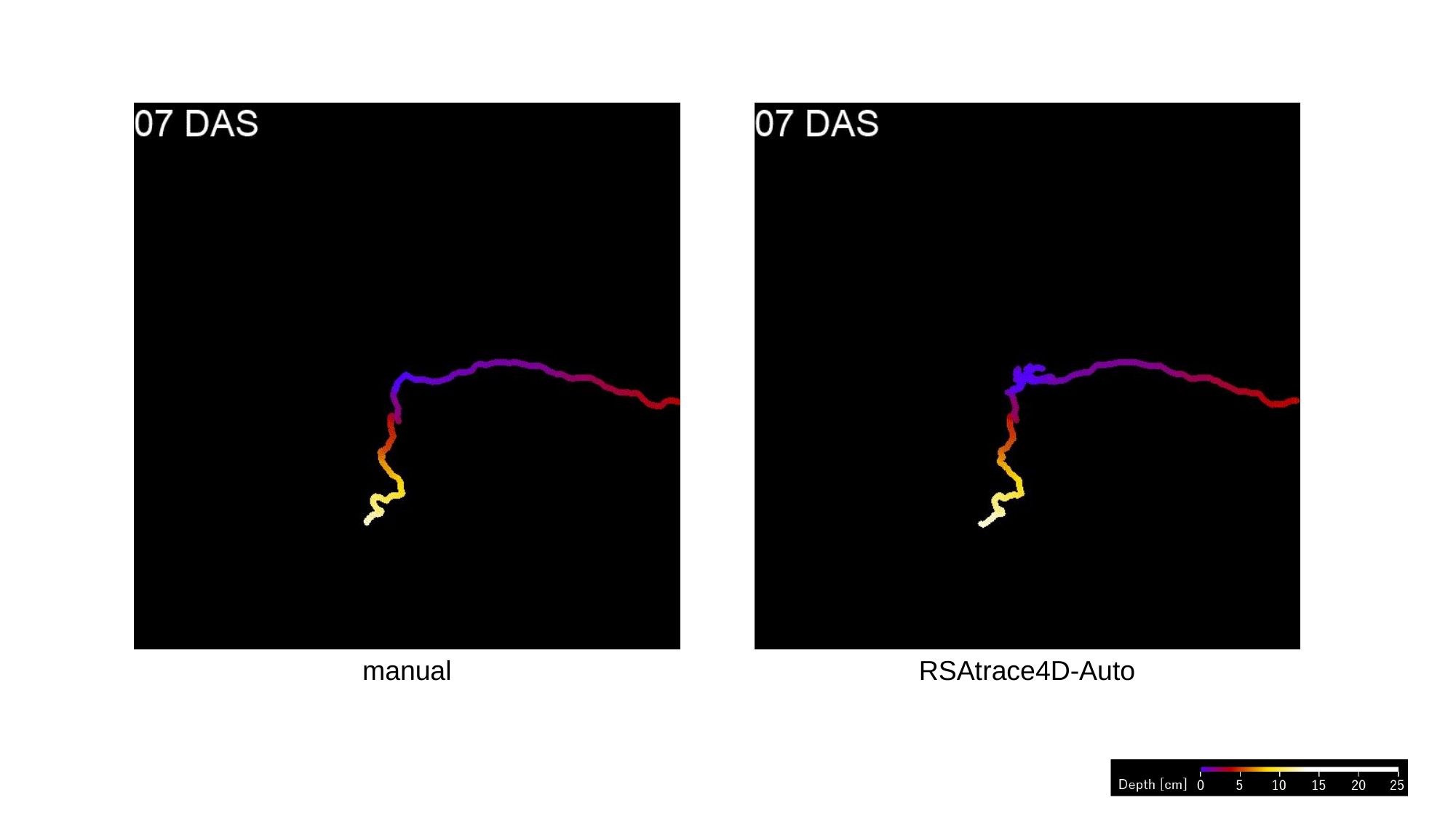

manual
RSAtrace4D-Auto

Supplement: Supplementary file 3 — Additional file 3: Movie S3. Animations of 21 top-view projections of rice RSA computed using RSA vectors generated by backward prediction. The color scale indicates the depth. [file 13007_2022_968_MOESM3_ESM.pptx]
