# Supplementary material for: Four-dimensional measurement of root system development using time-series three-dimensional volumetric data analysis by backward prediction
Source: Plant Methods. 2022 Dec 9;18:133. doi: 10.1186/s13007-022-00968-x (PMC9733169; doi:10.1186/s13007-022-00968-x)
Supplement: Supplementary file 2 — Additional file 2: Movie S2. Animations of backward prediction results from 7 to 27 DAS. [file 13007_2022_968_MOESM2_ESM.pptx]

## Slide 1
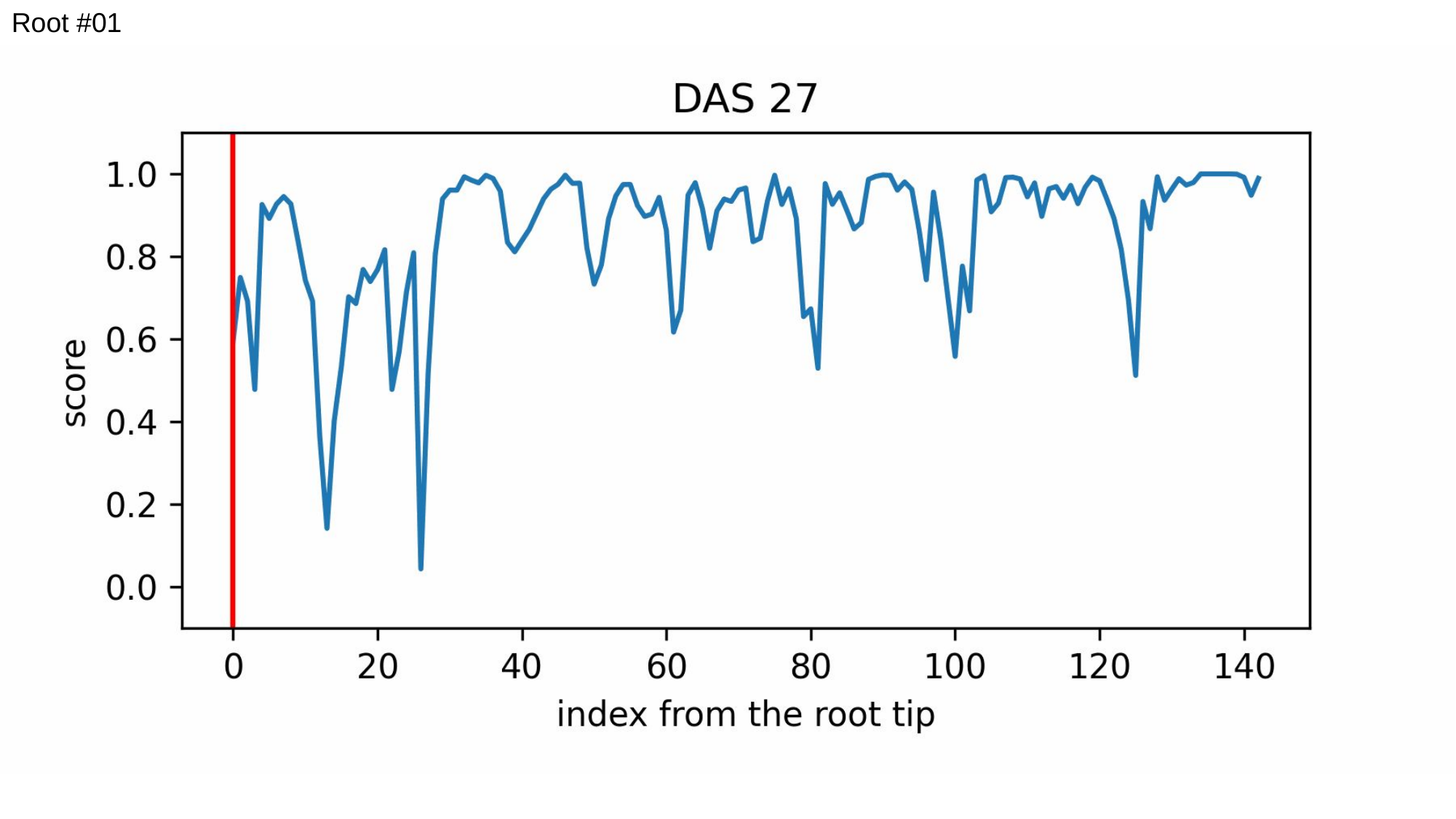

Root #01

## Slide 2
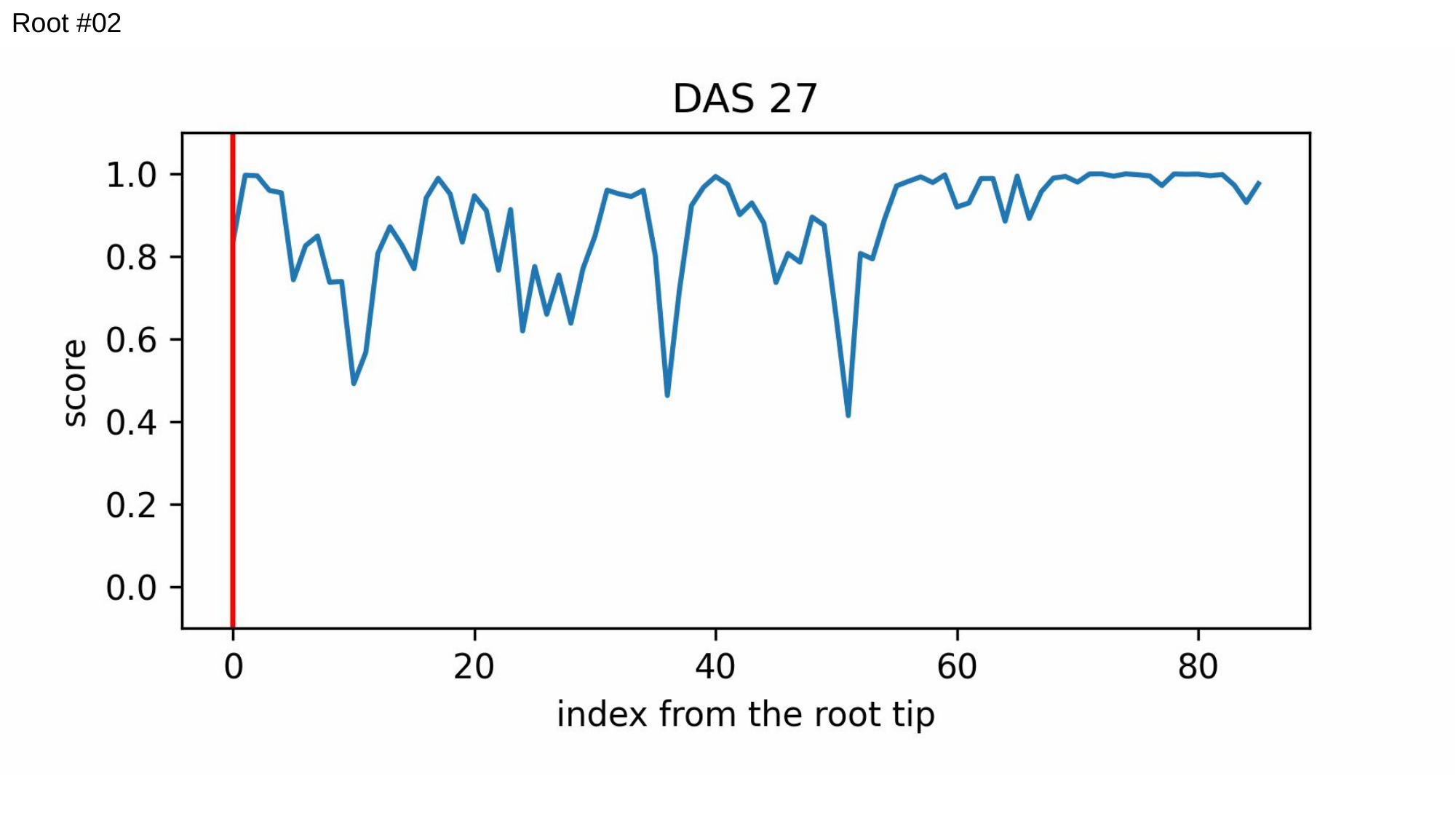

Root #02

## Slide 3
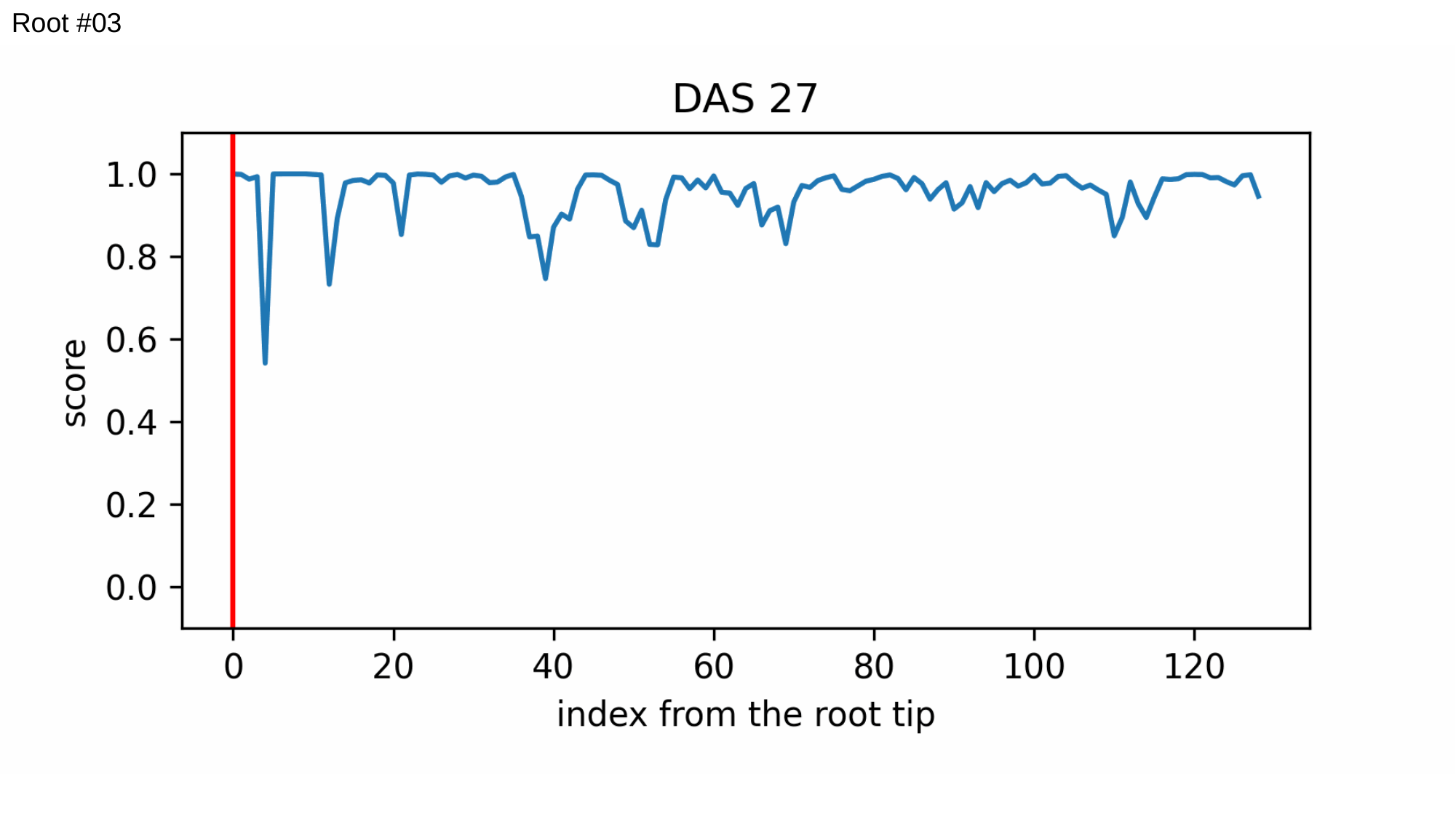

Root #03

## Slide 4
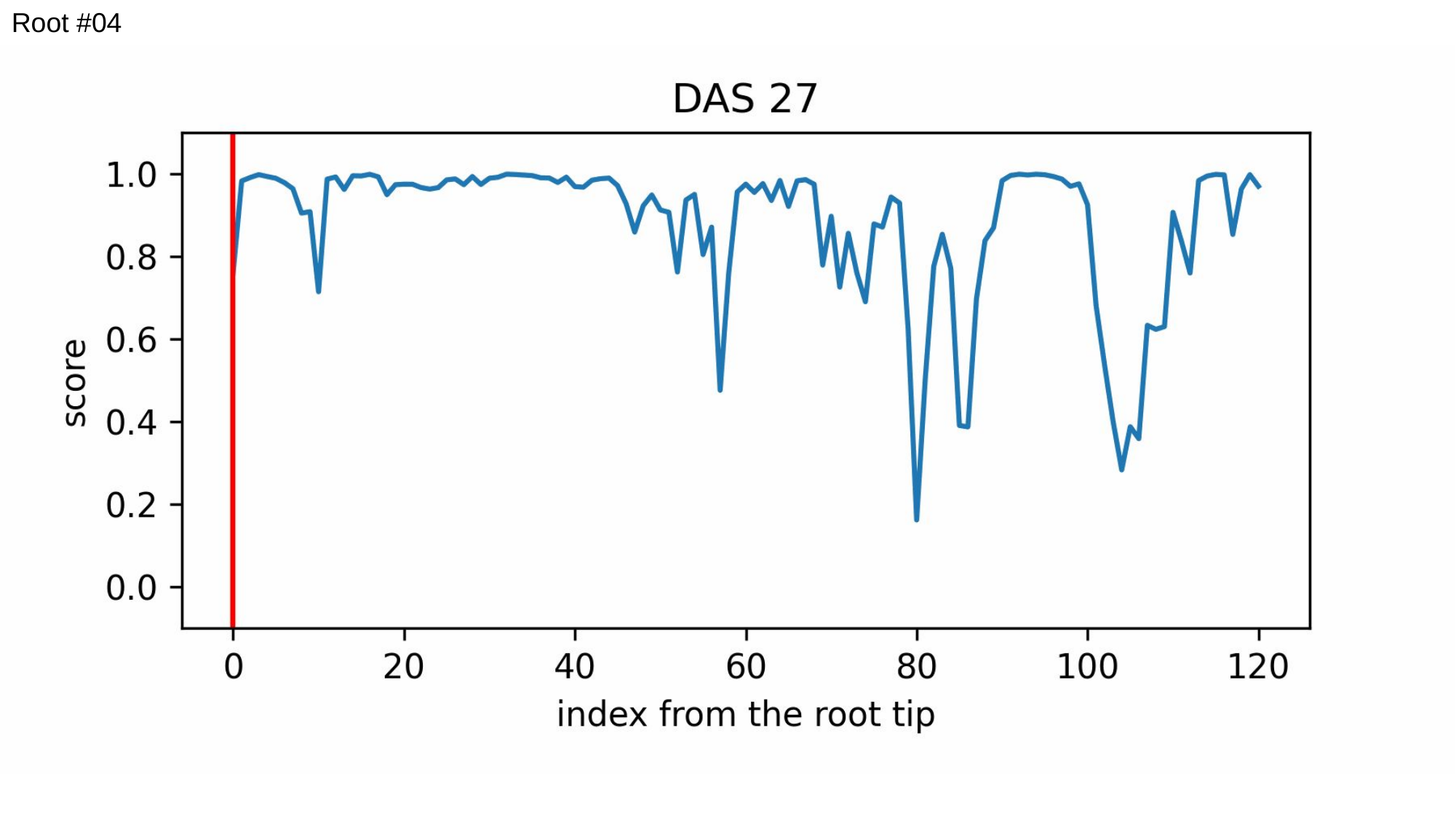

Root #04

## Slide 5
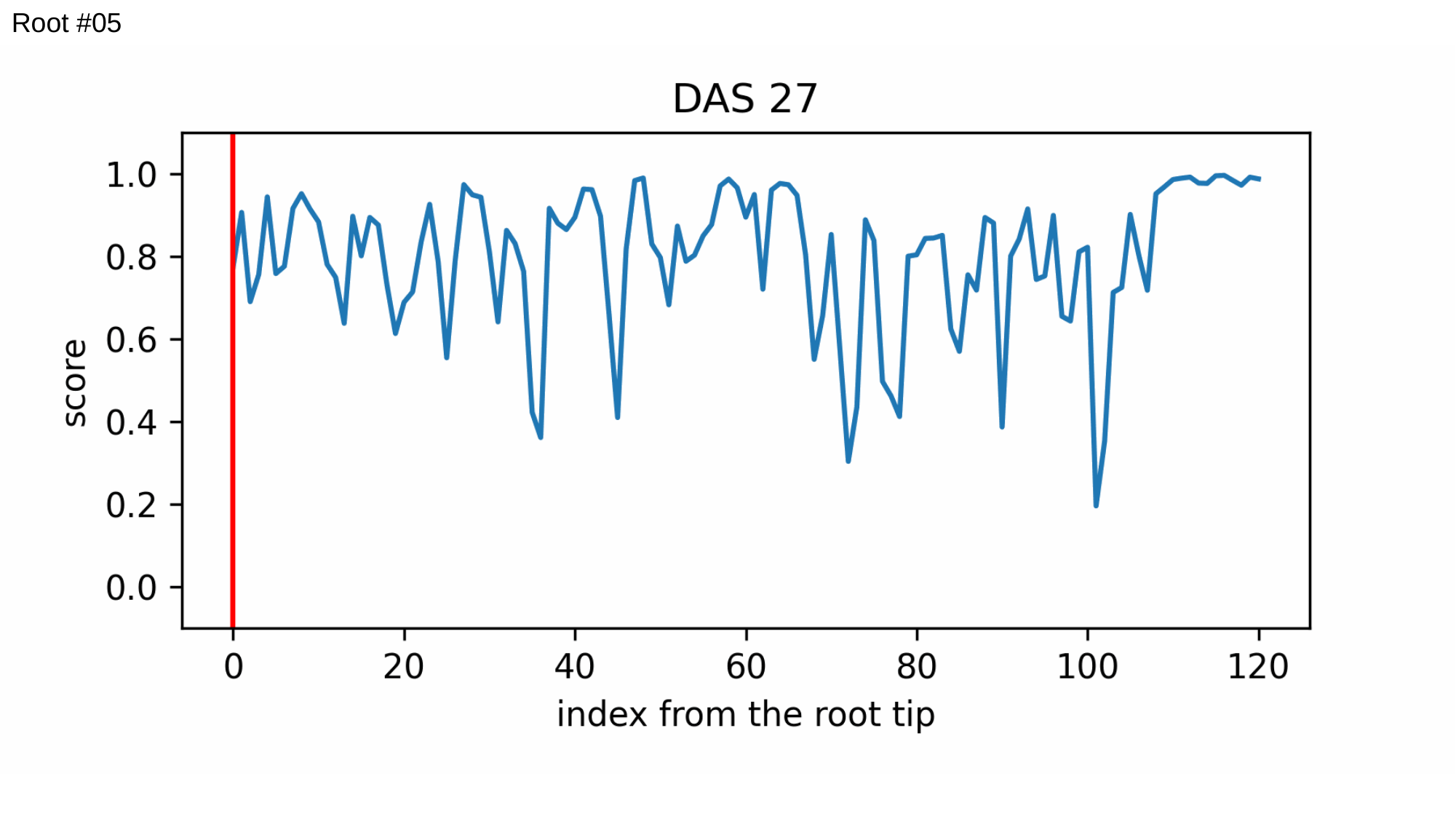

Root #05

## Slide 6
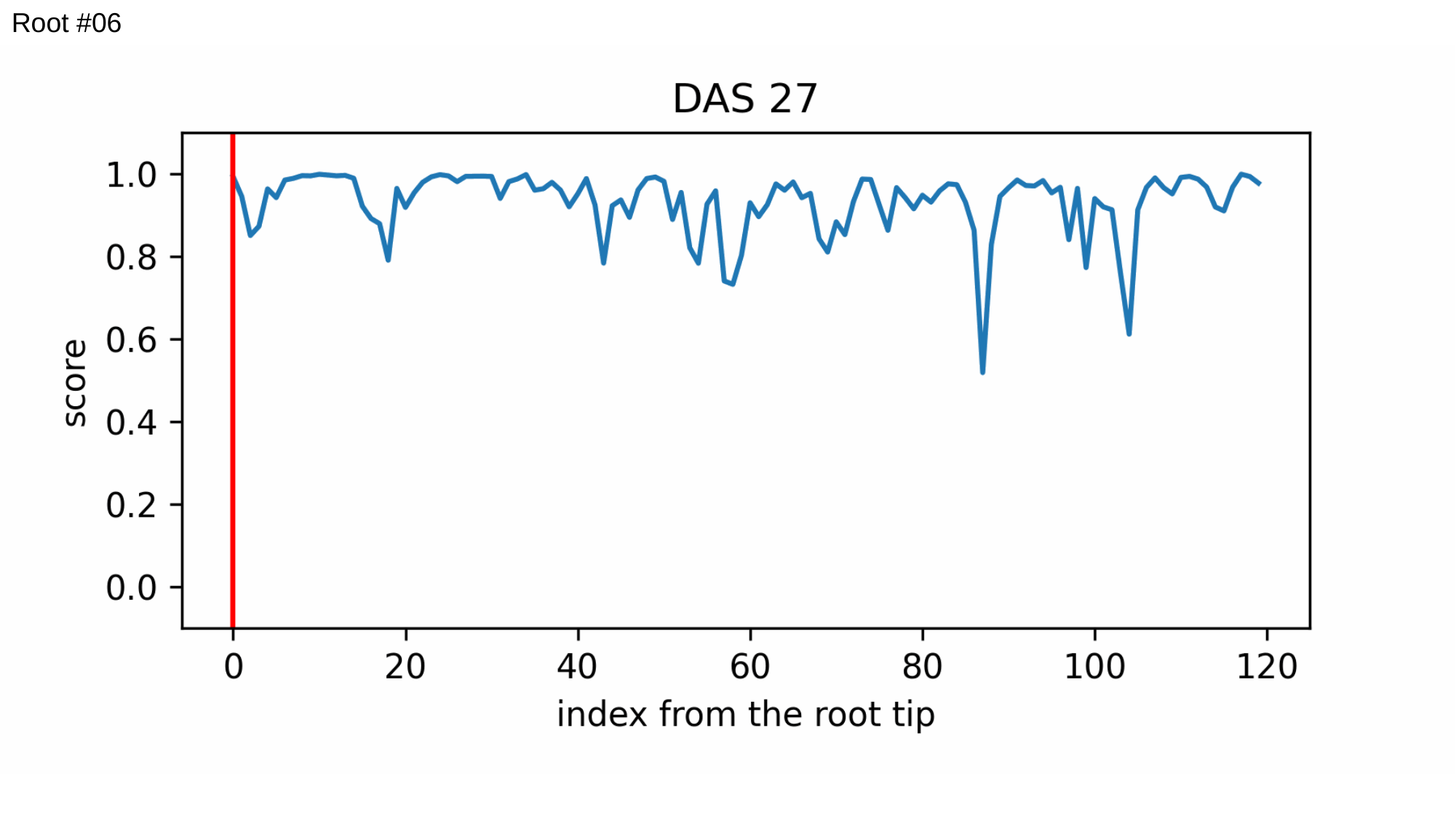

Root #06

## Slide 7
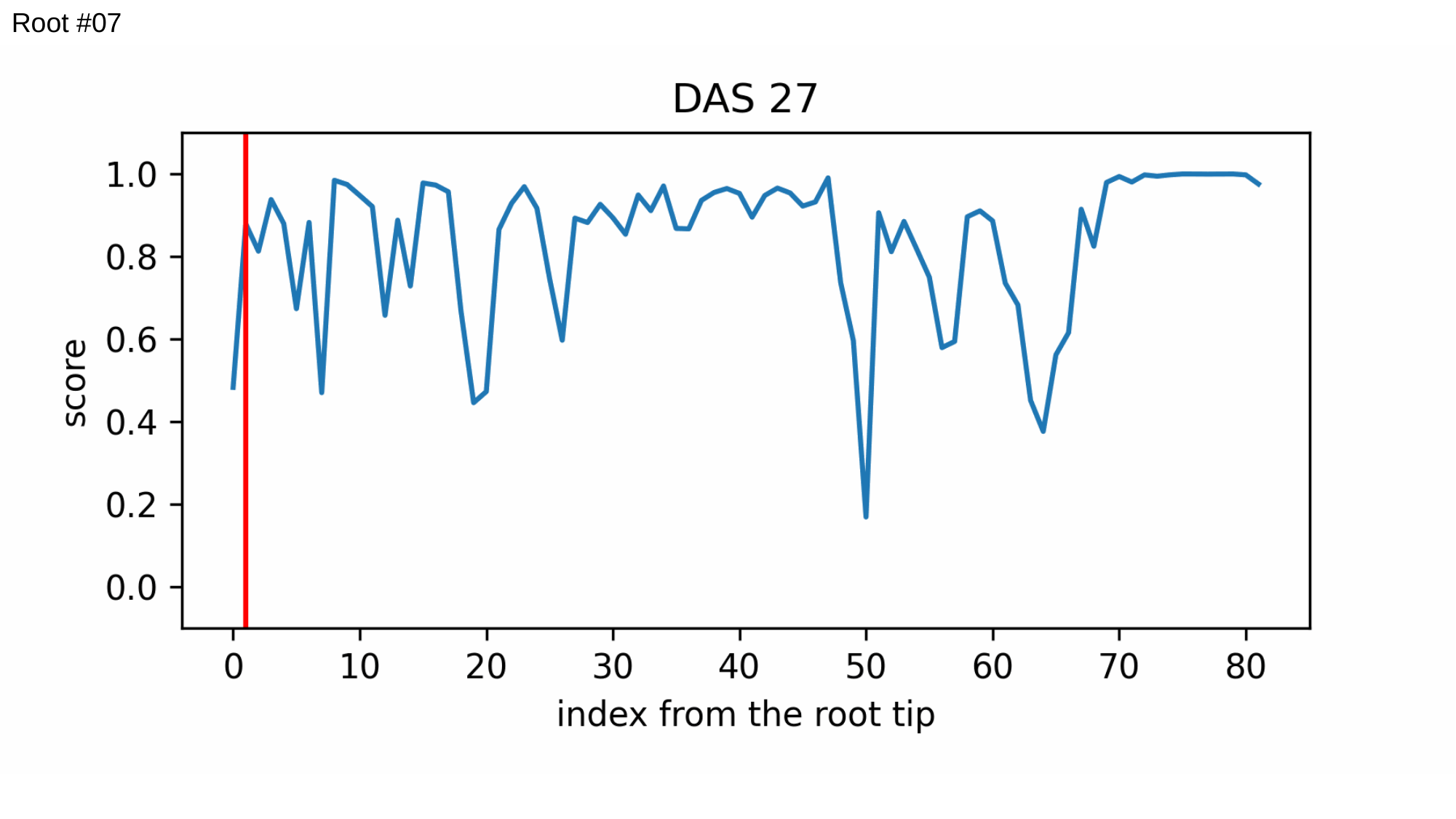

Root #07

## Slide 8
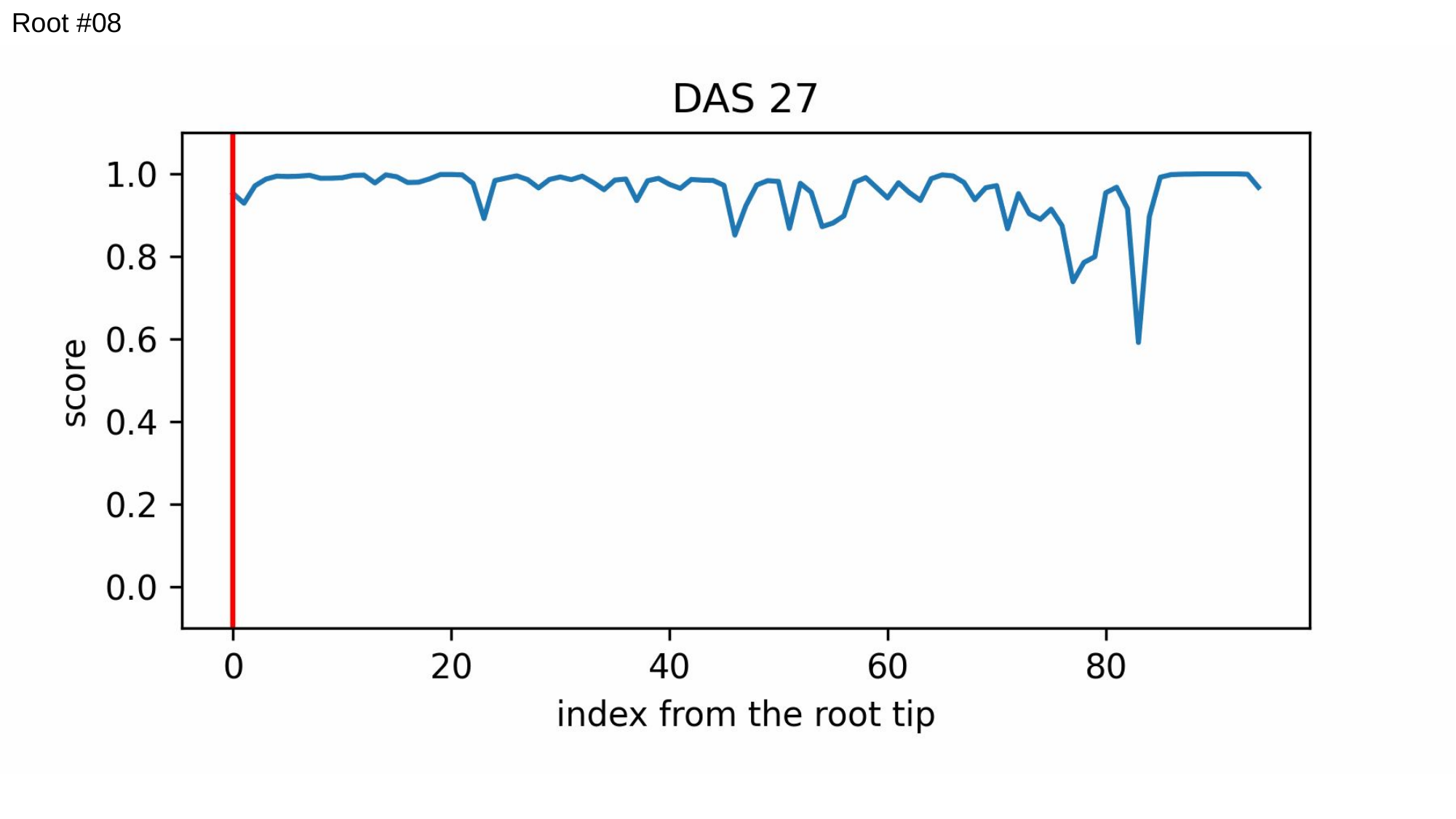

Root #08

## Slide 9
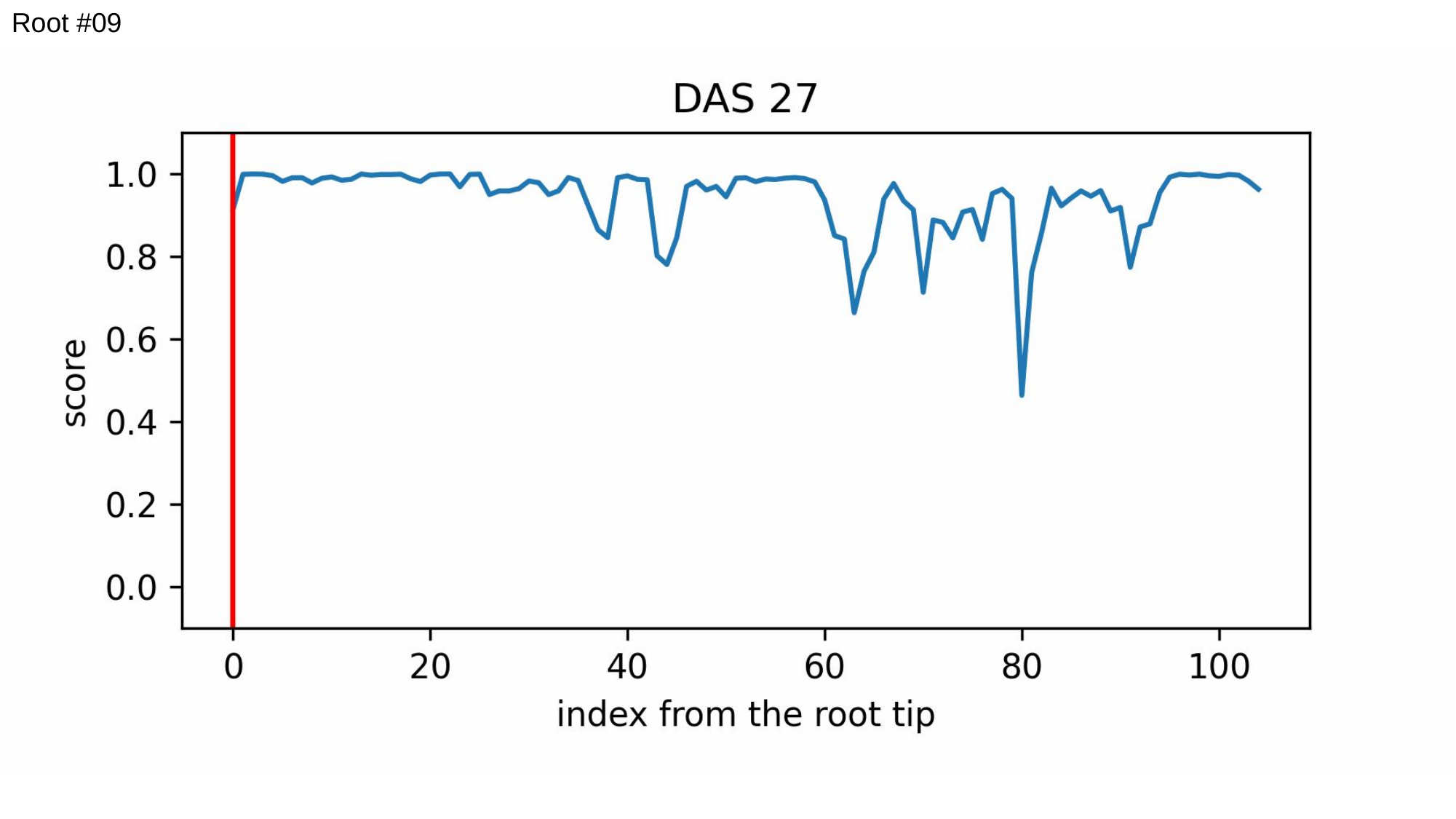

Root #09

## Slide 10
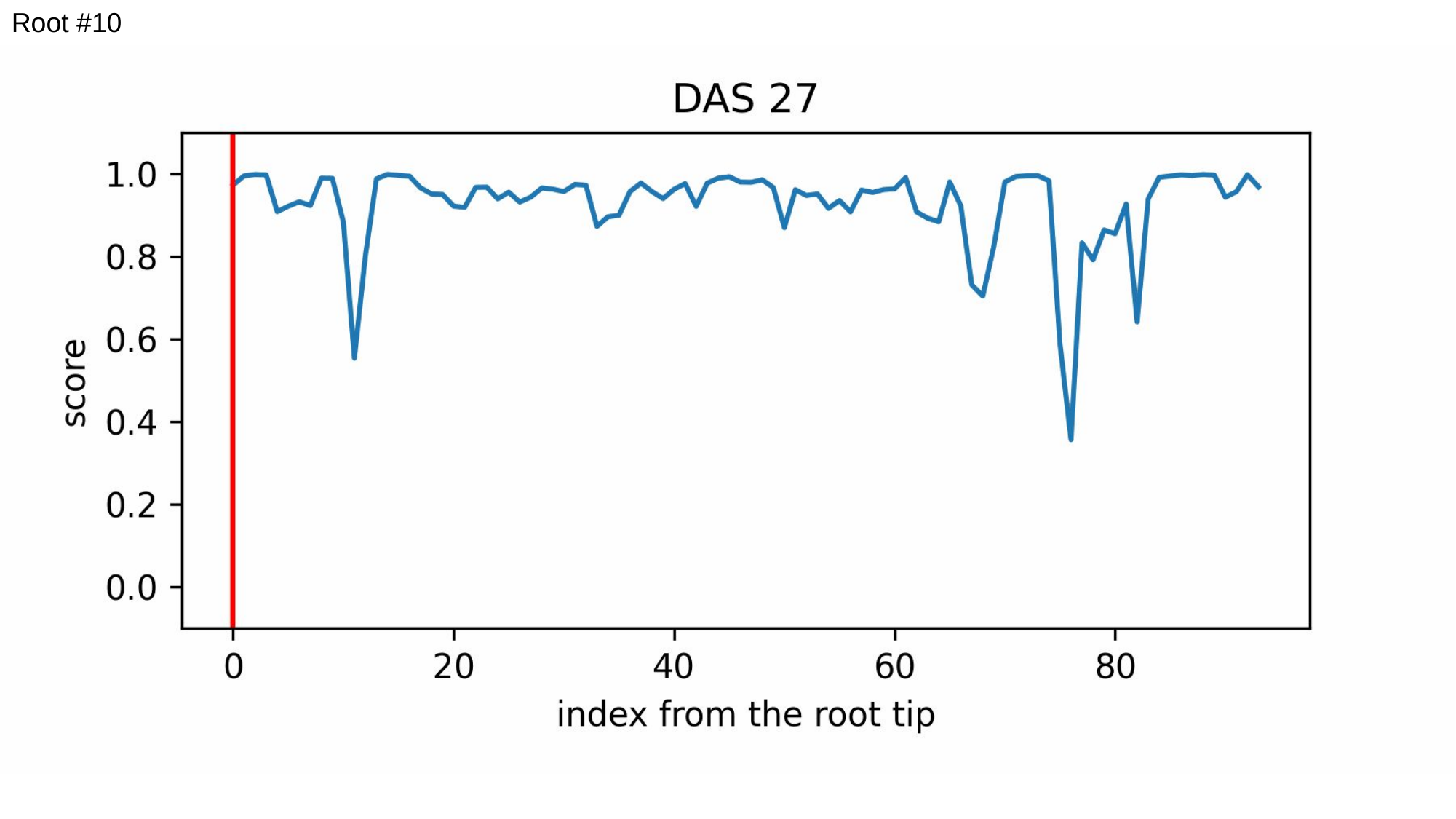

Root #10

## Slide 11
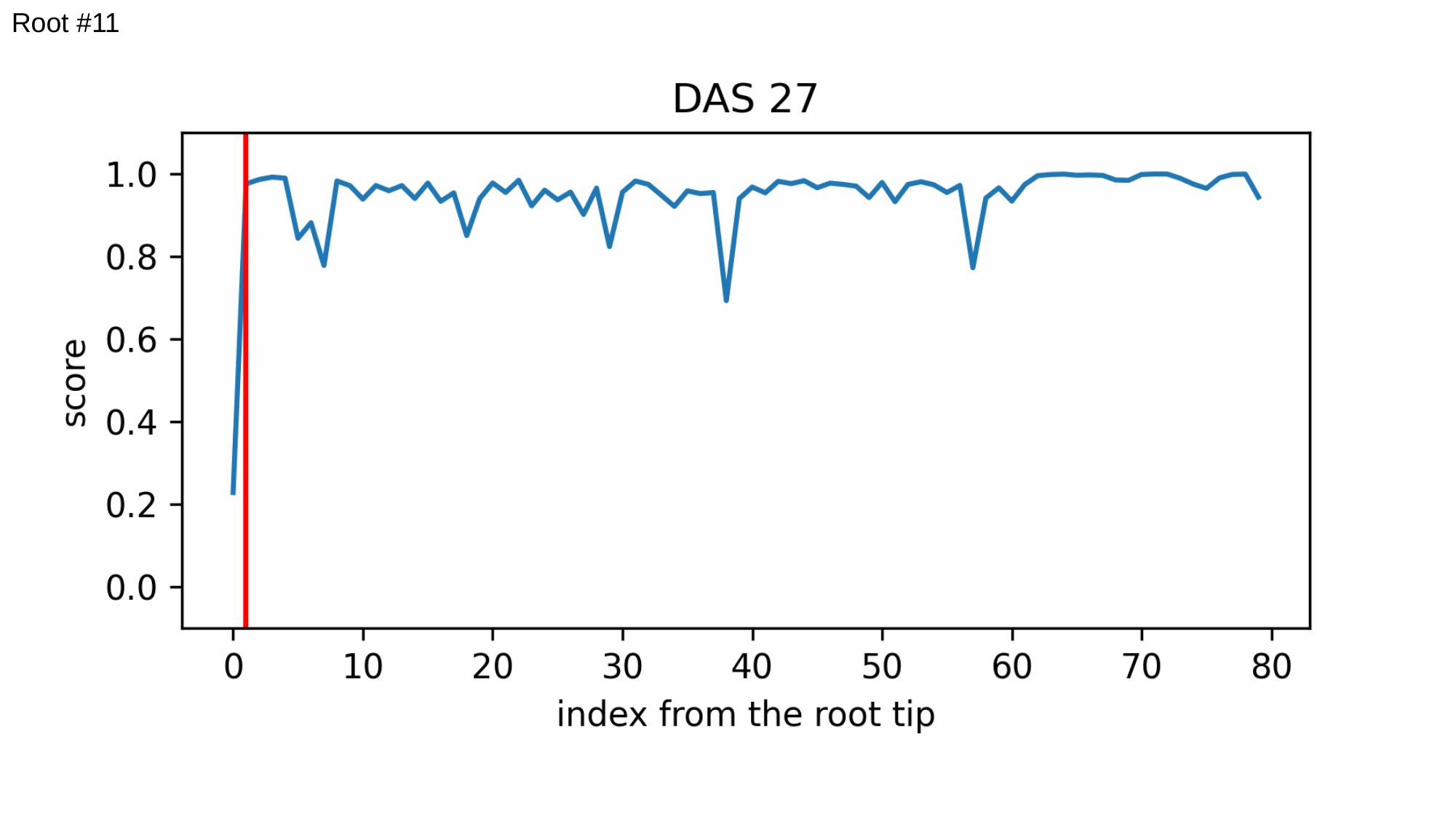

Root #11

## Slide 12
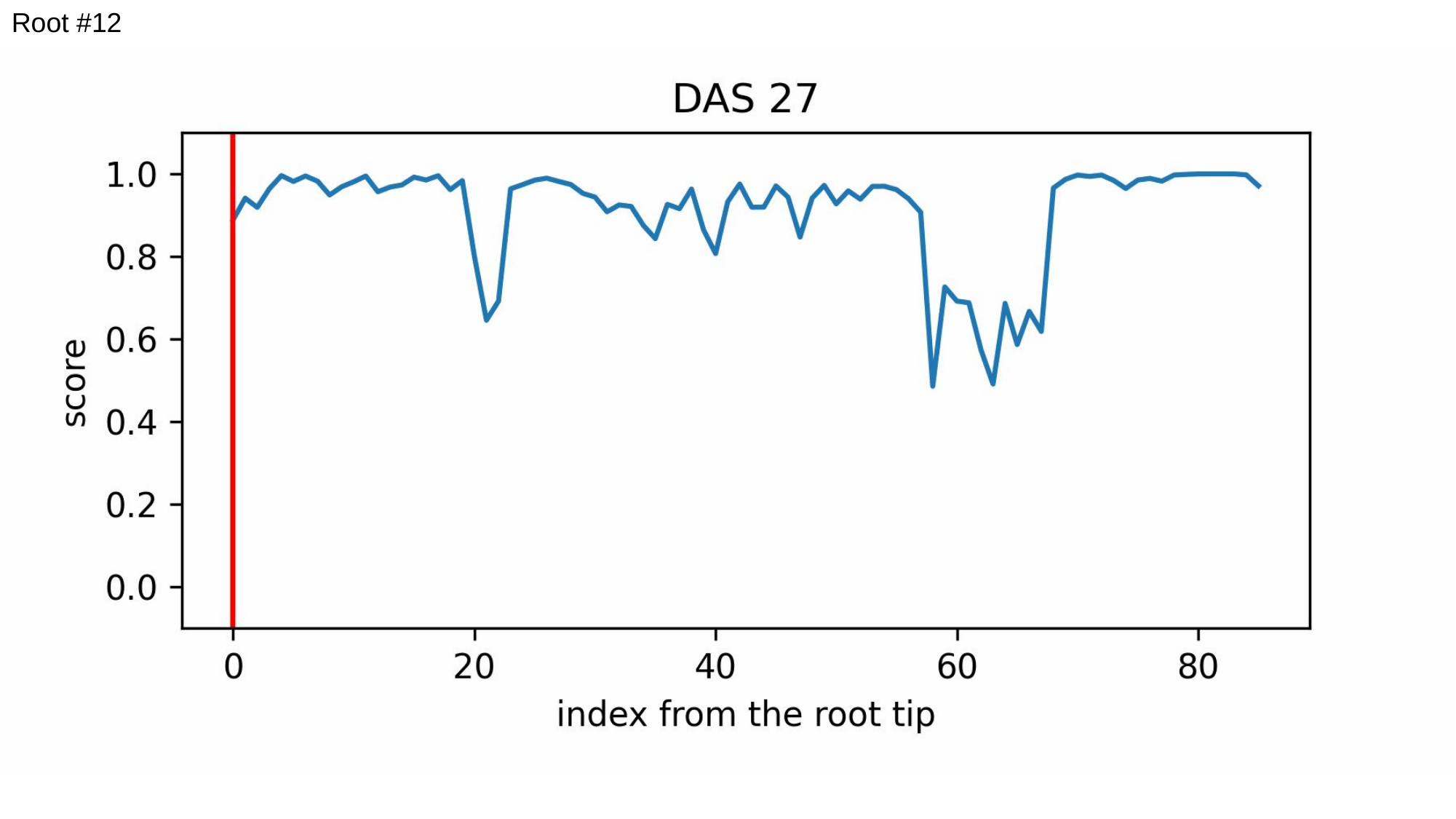

Root #12

## Slide 13
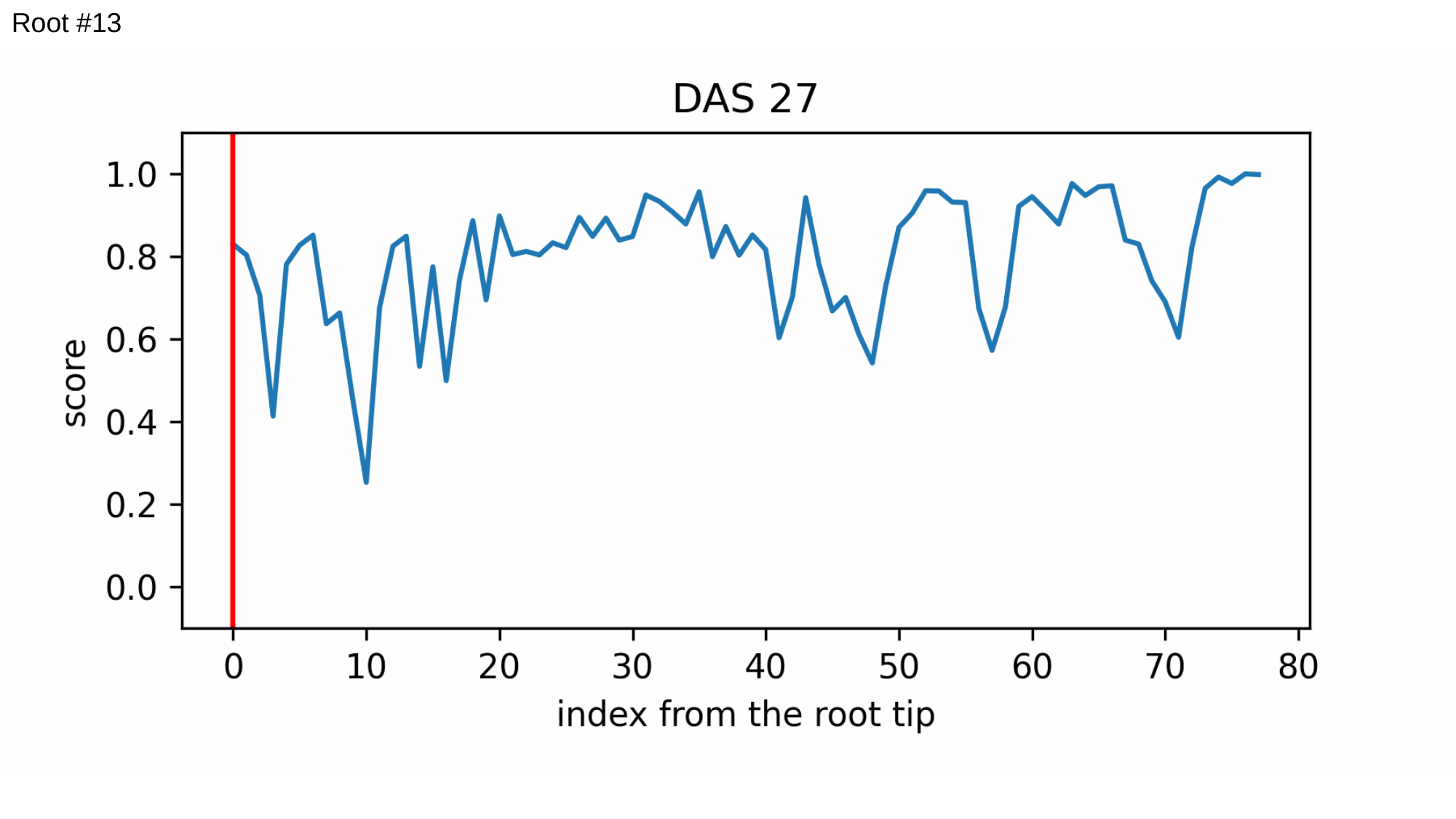

Root #13

## Slide 14
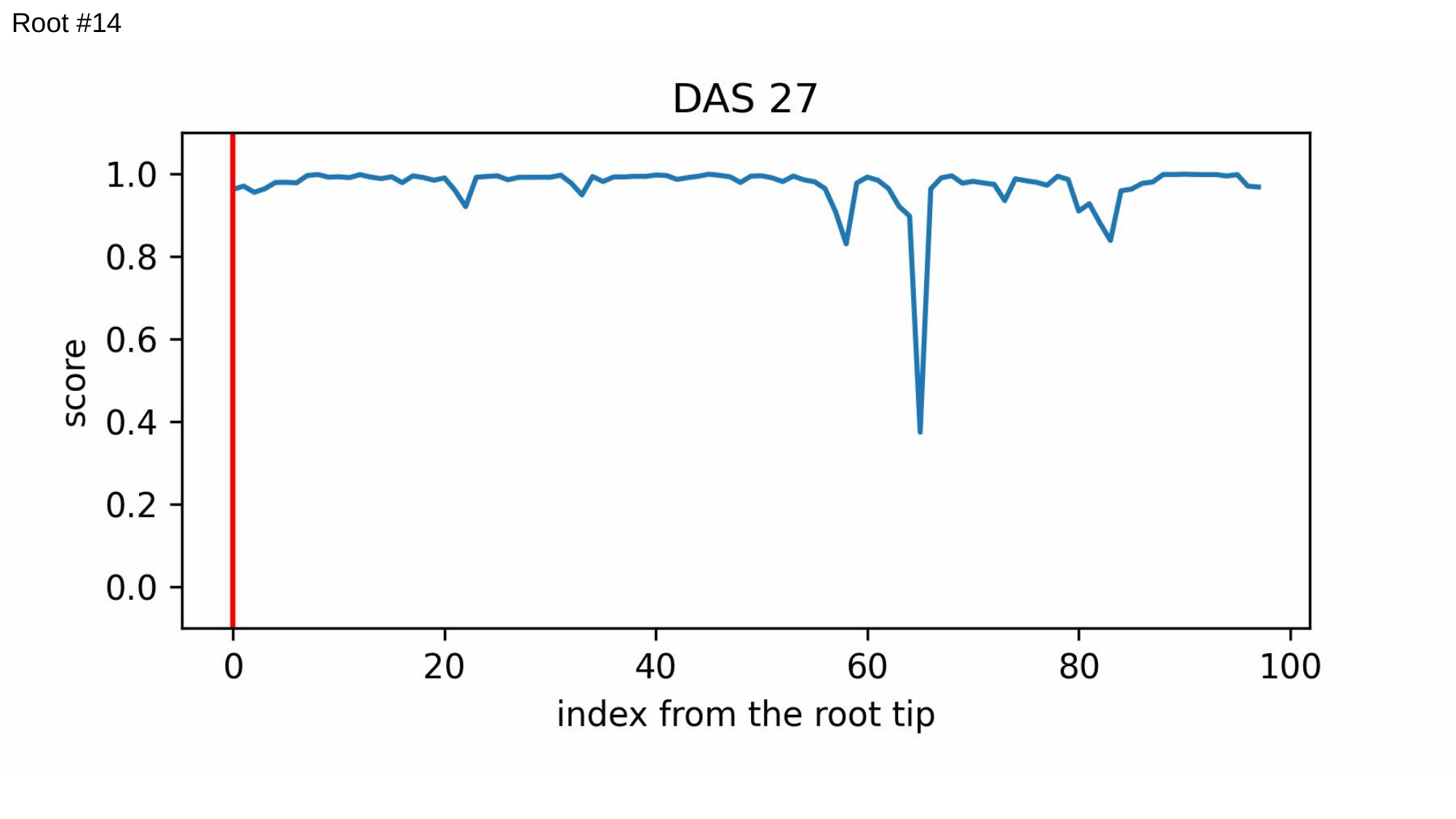

Root #14

## Slide 15
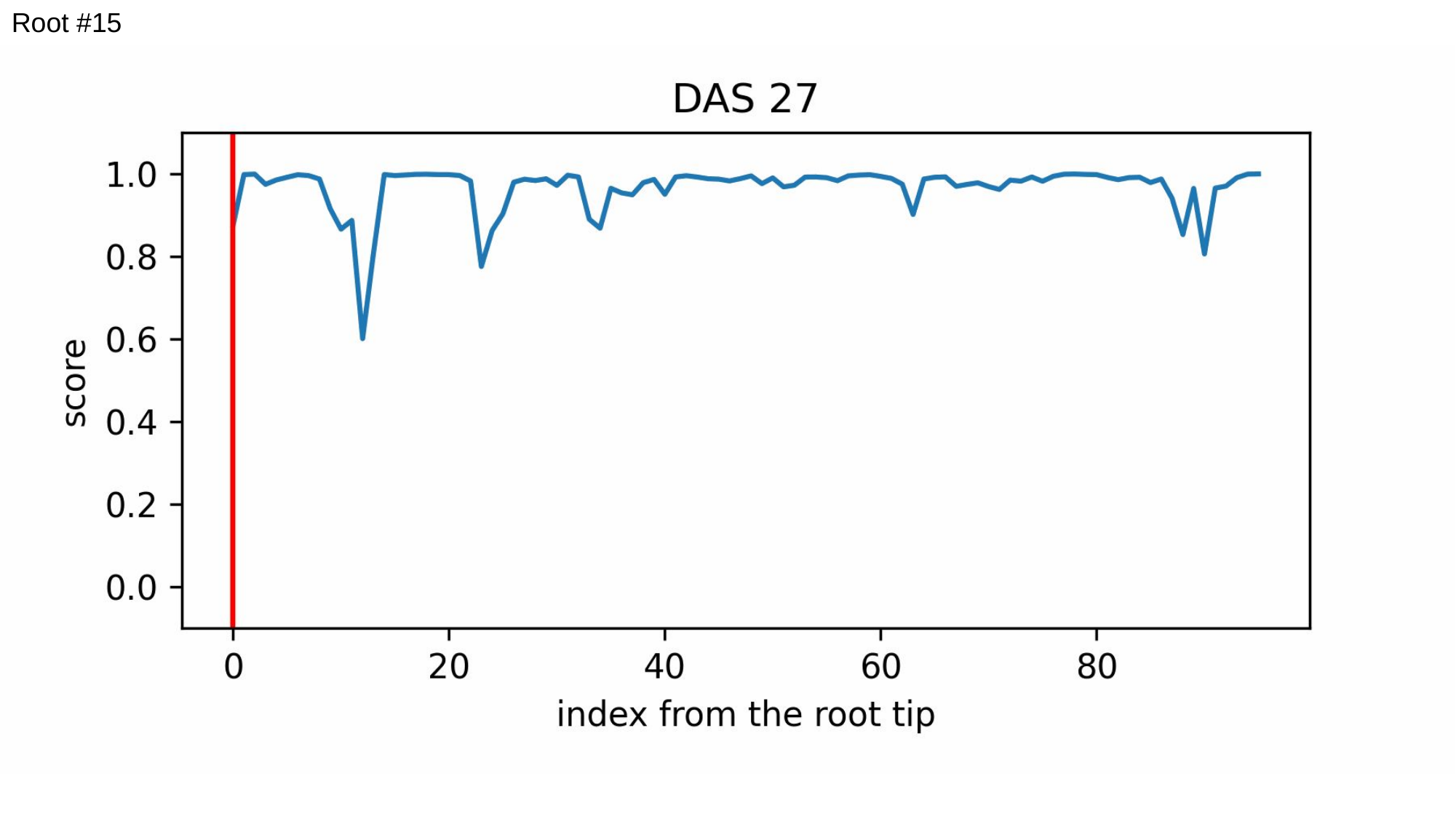

Root #15

## Slide 16
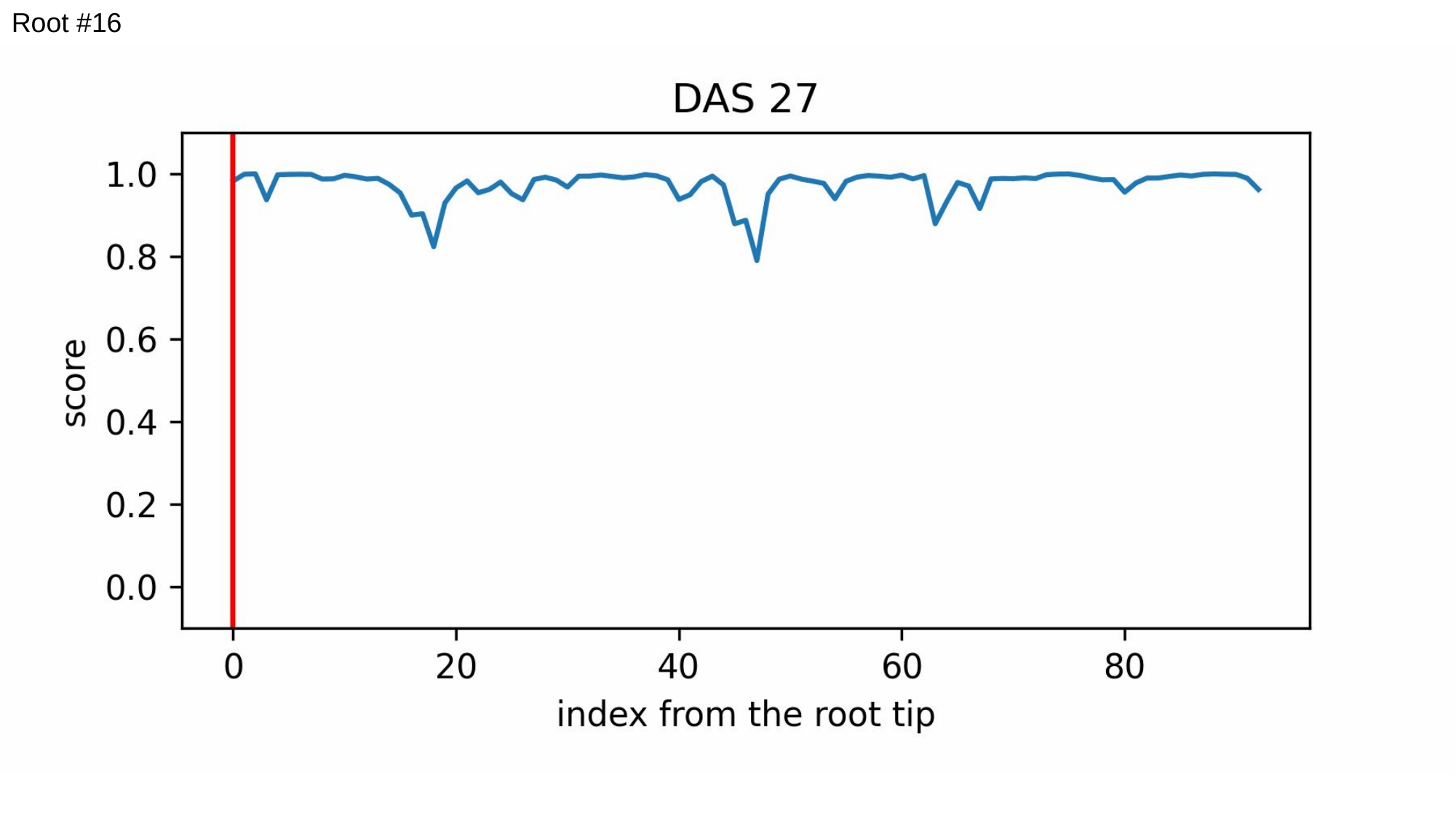

Root #16

## Slide 17
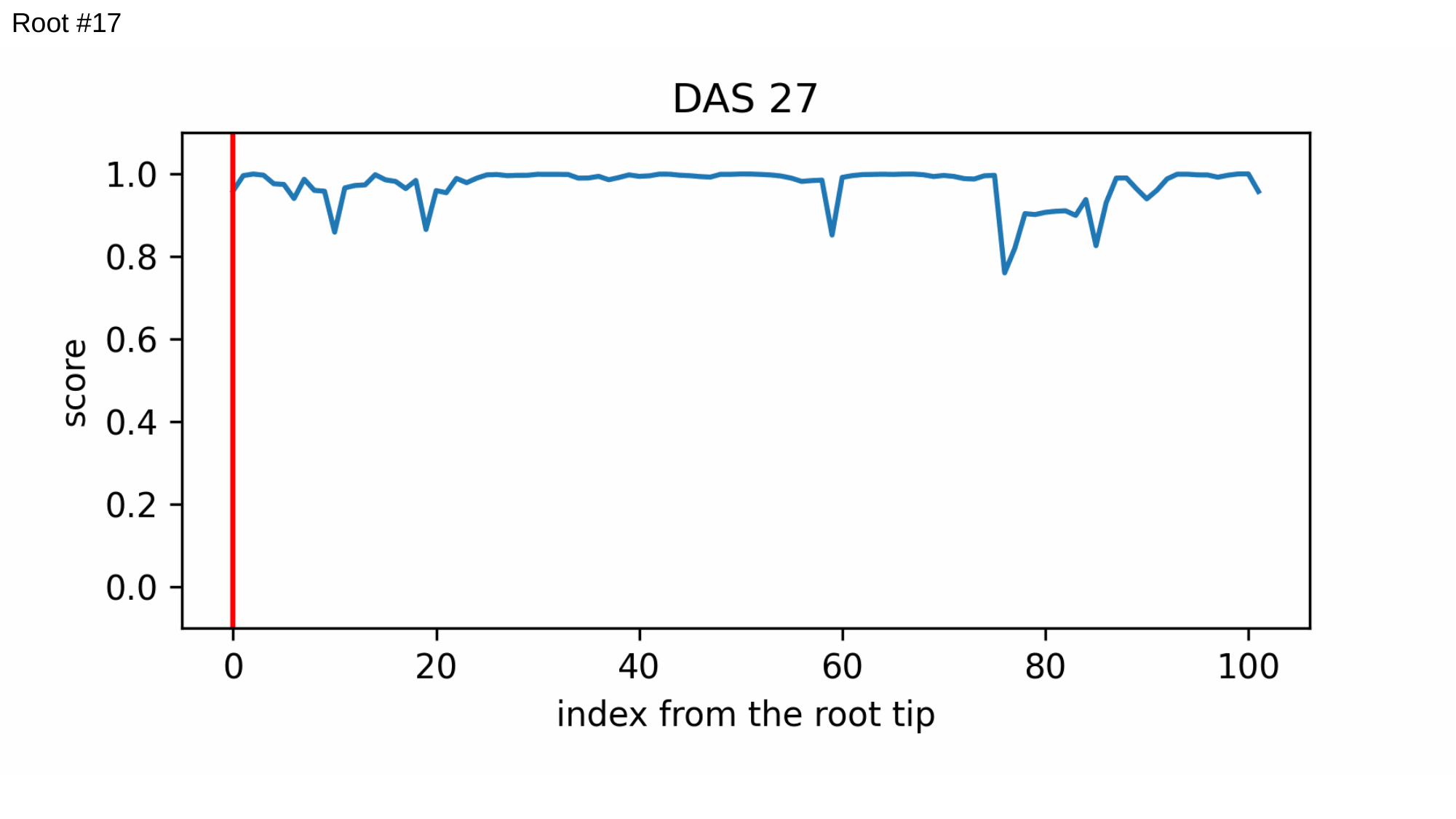

Root #17

## Slide 18
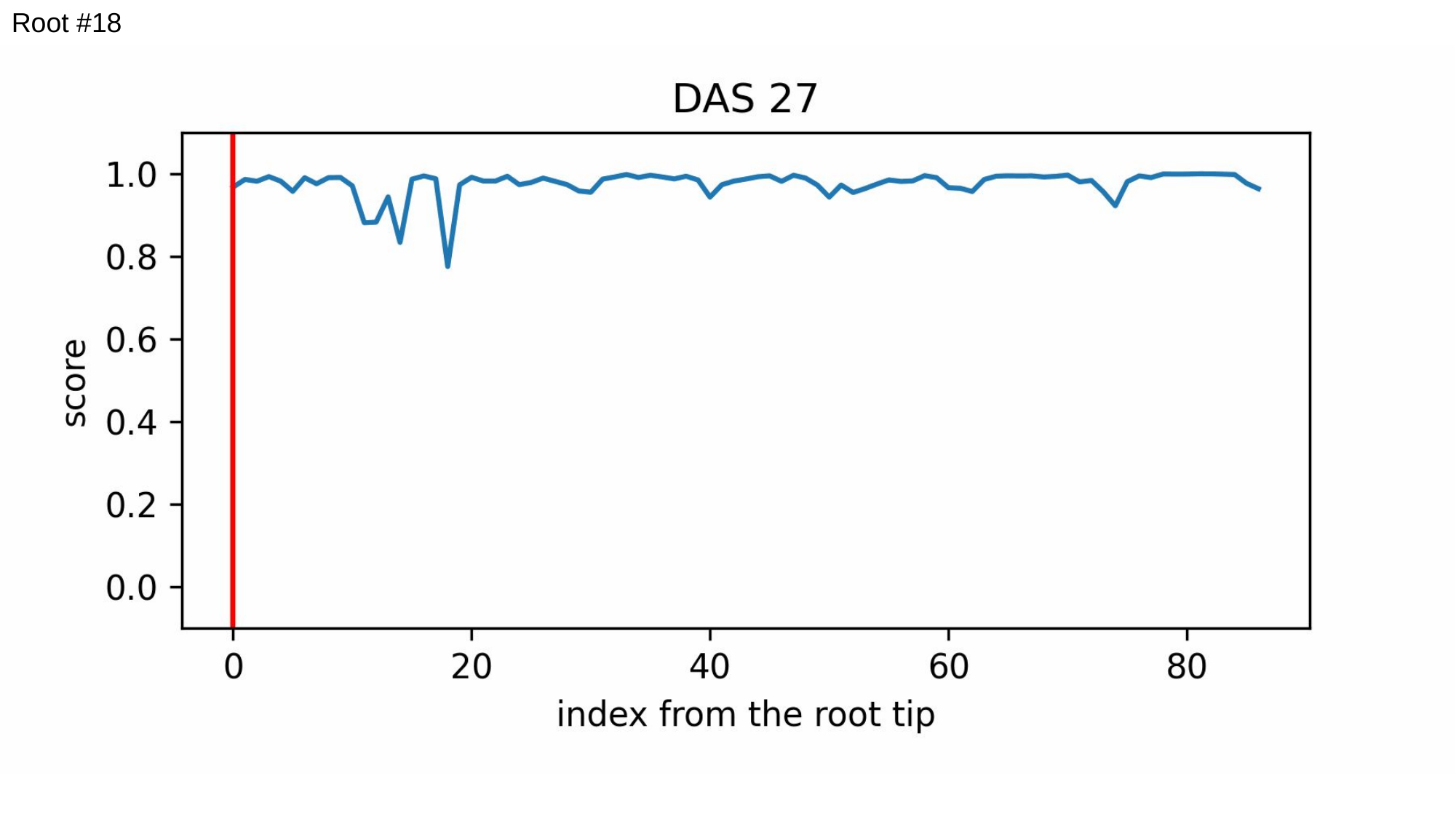

Root #18

## Slide 19
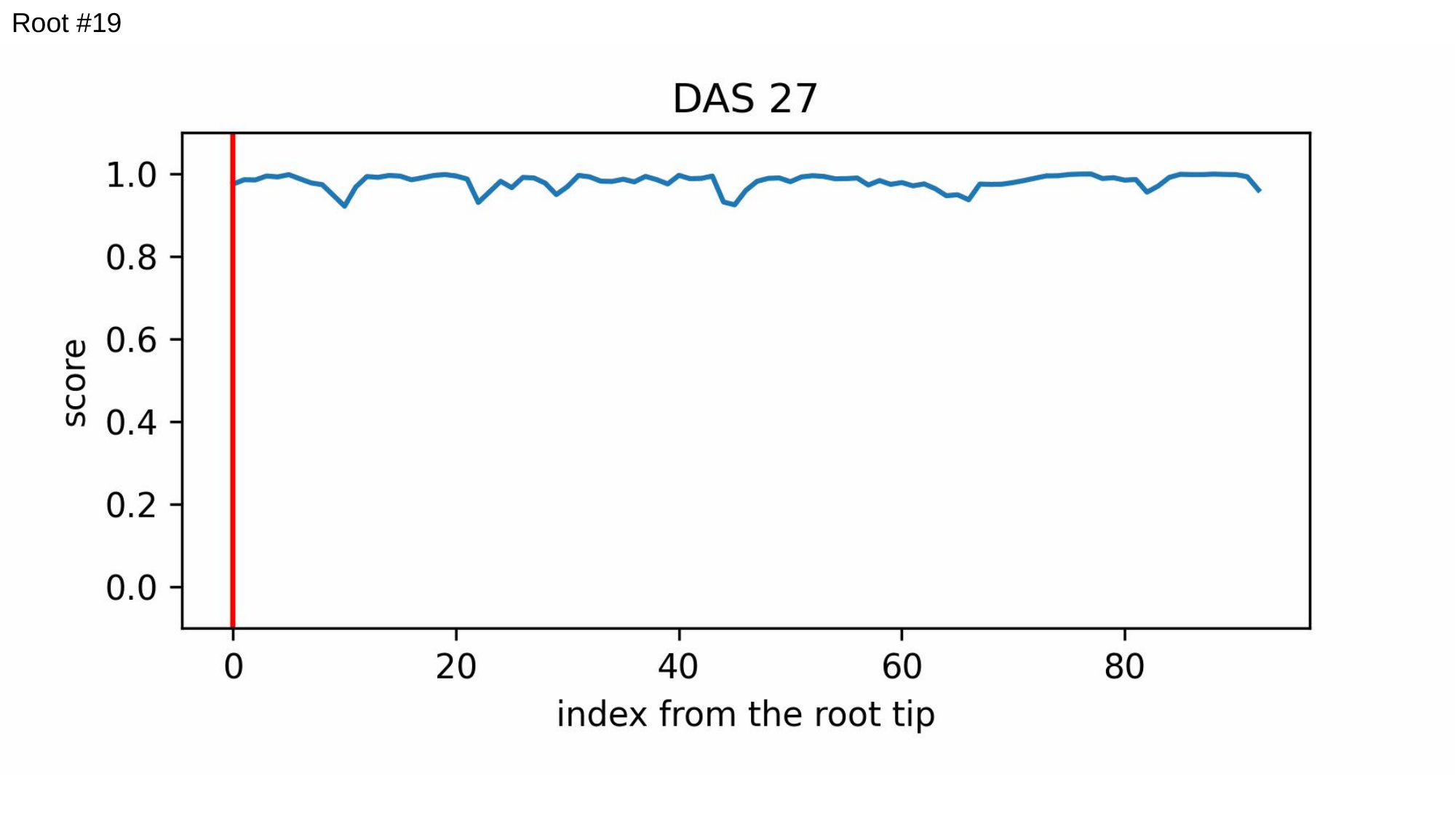

Root #19

## Slide 20
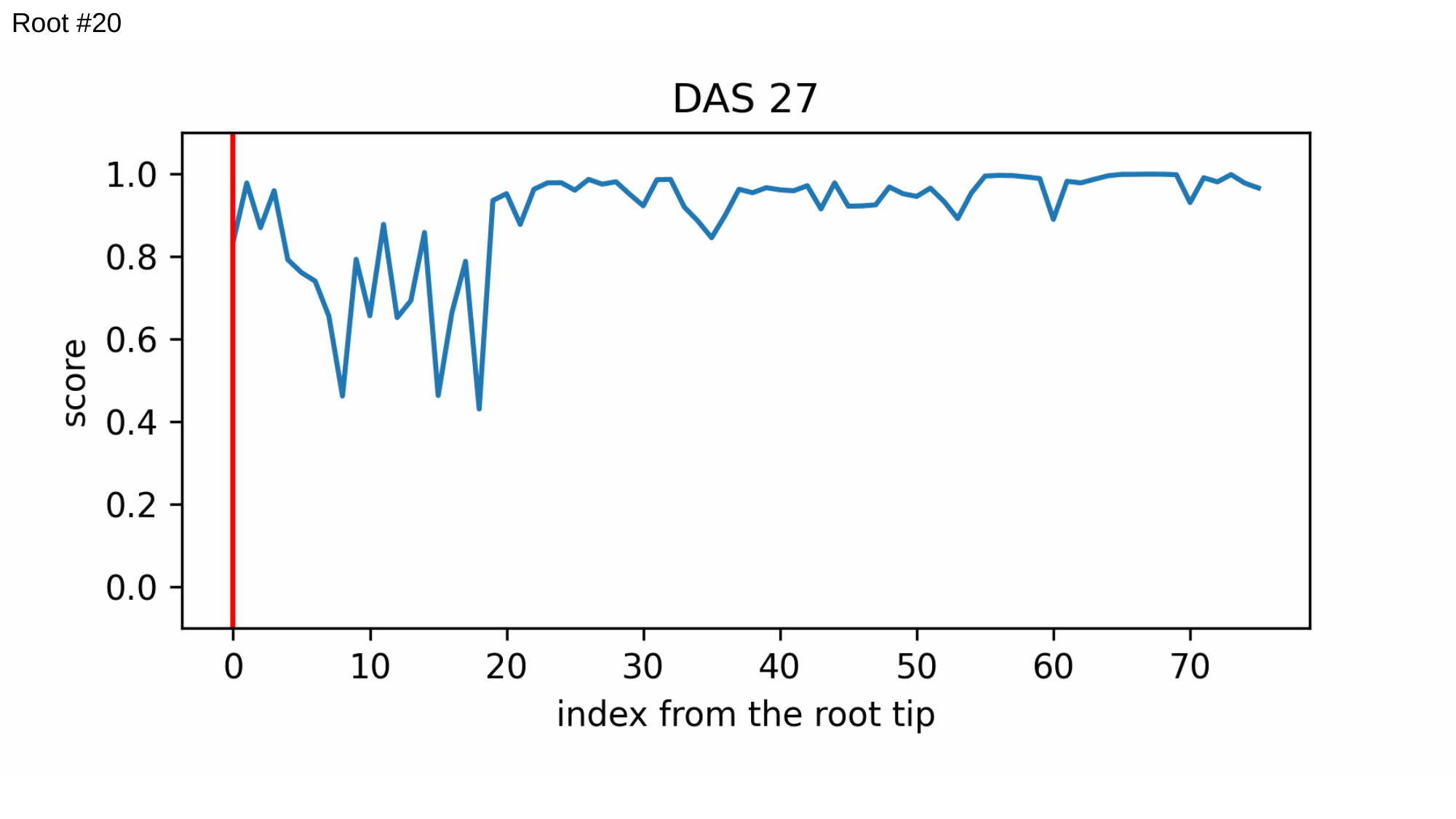

Root #20

## Slide 21
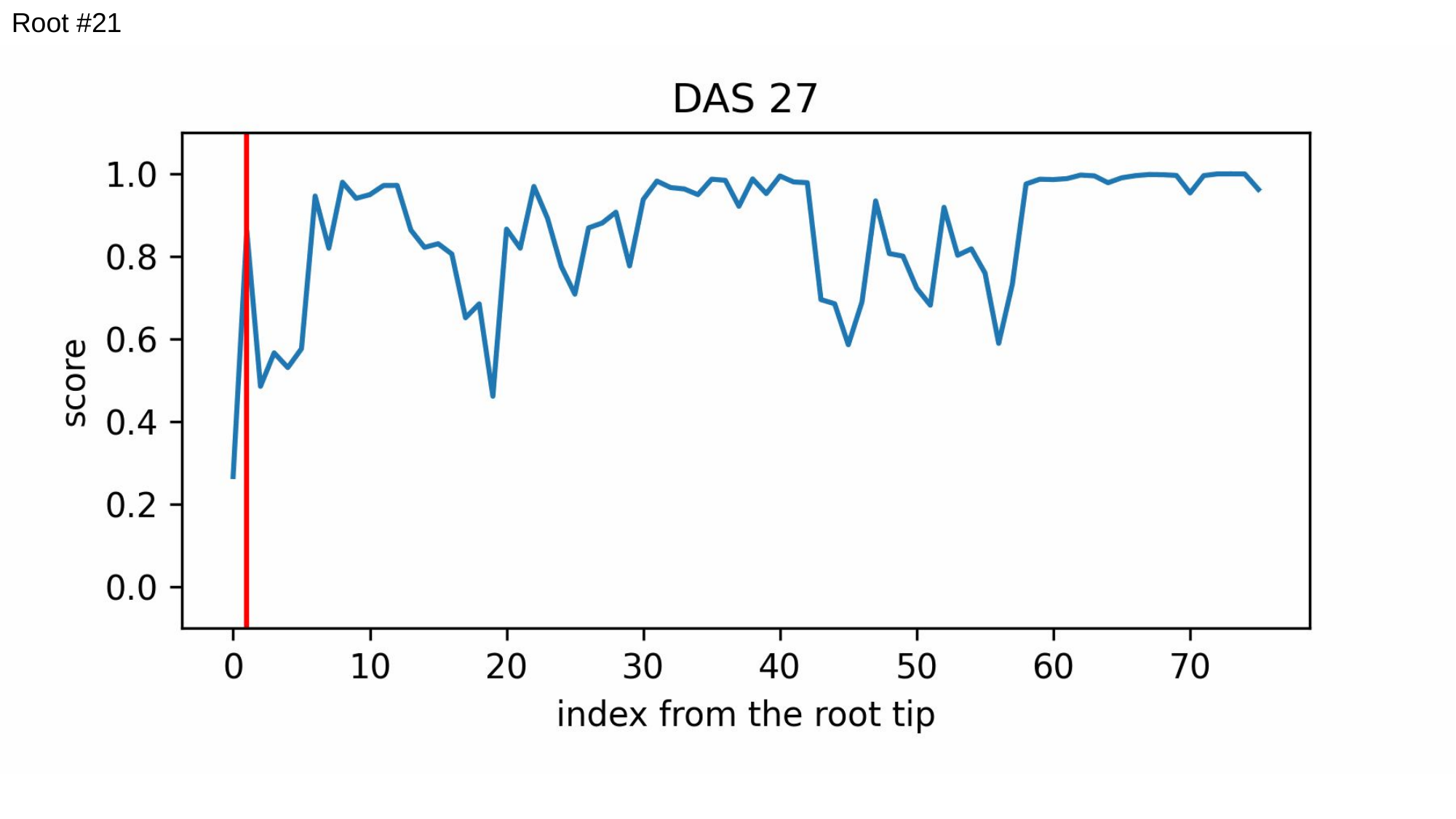

Root #21

## Slide 22
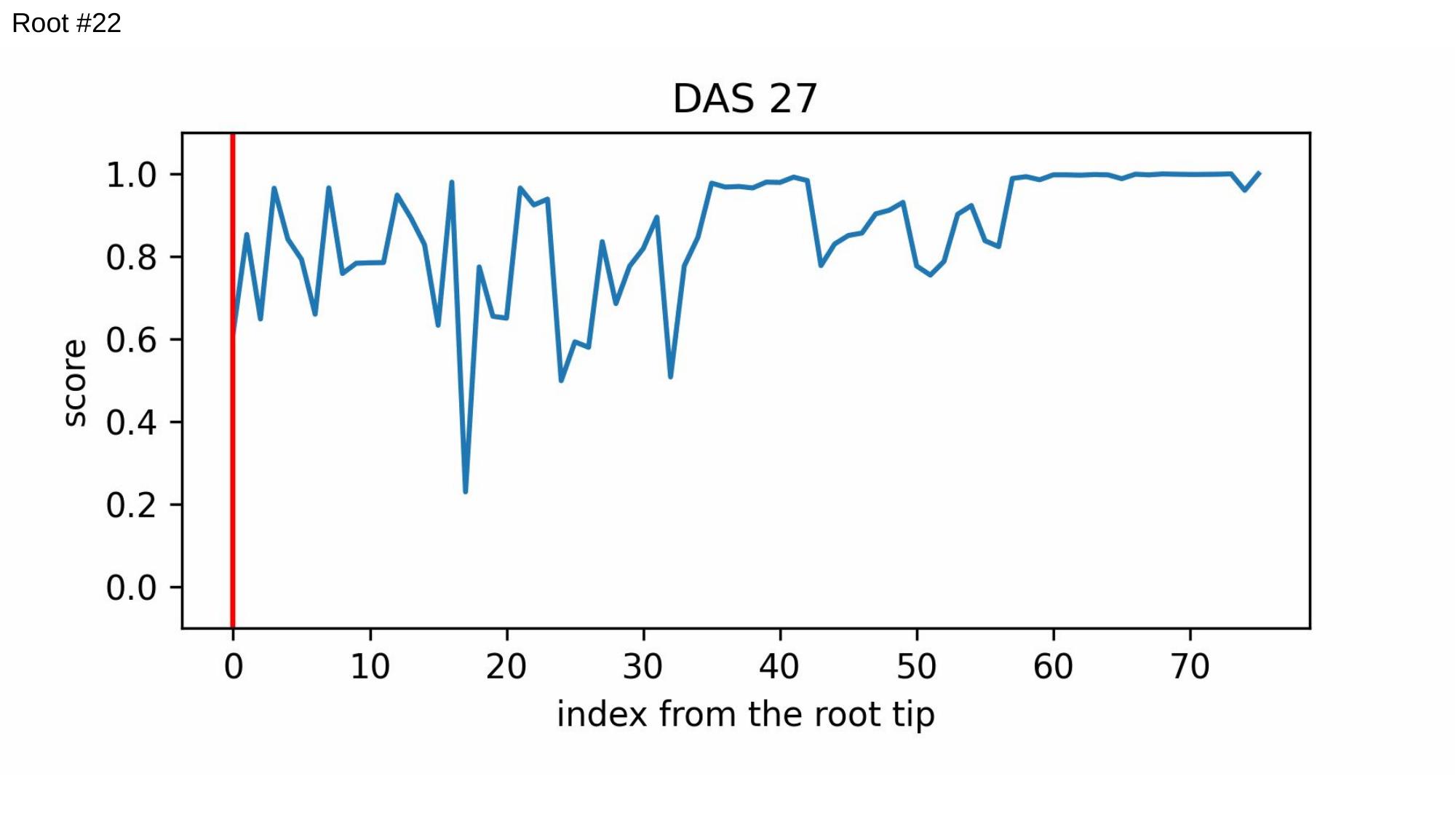

Root #22

## Slide 23
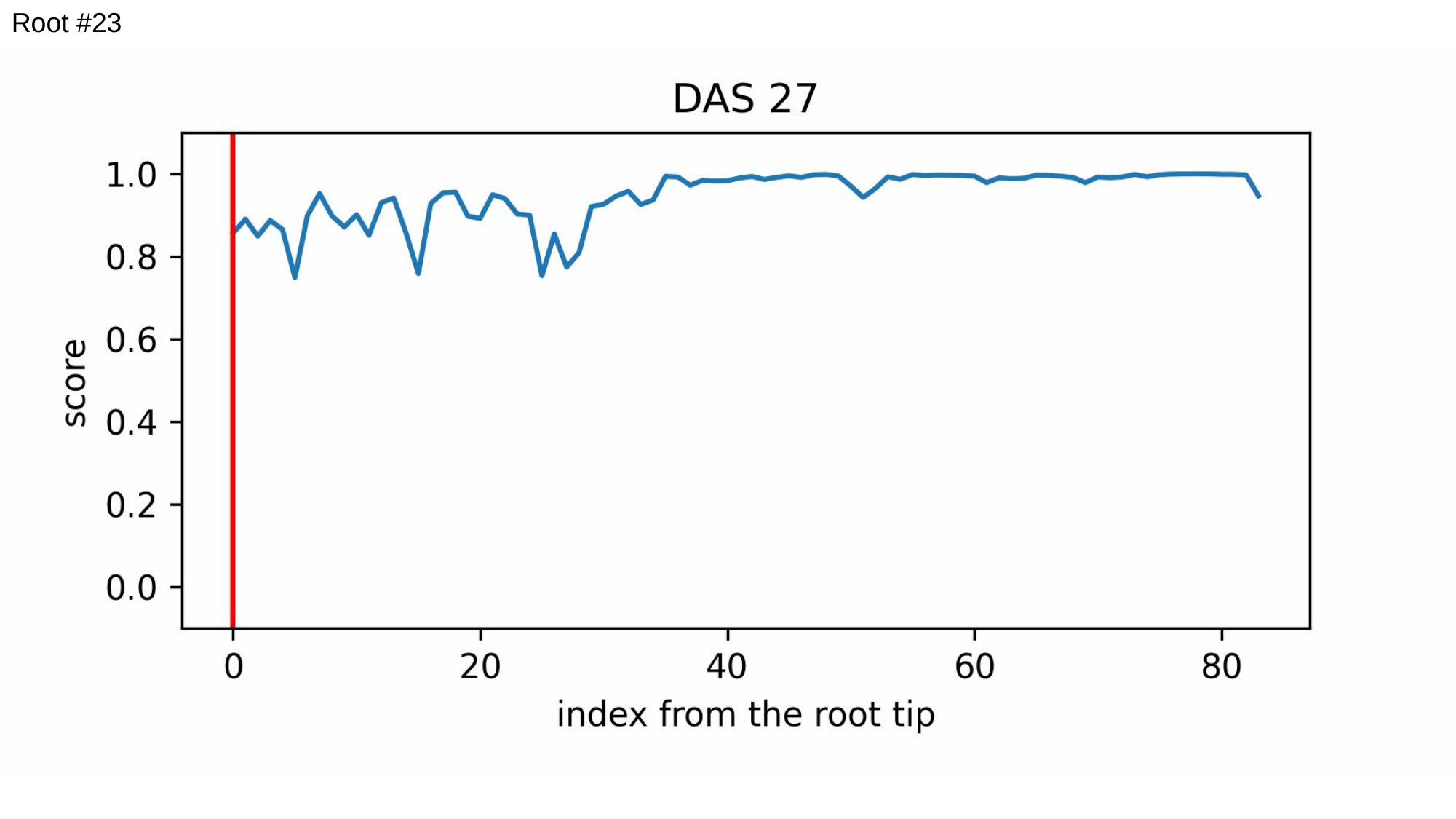

Root #23

## Slide 24
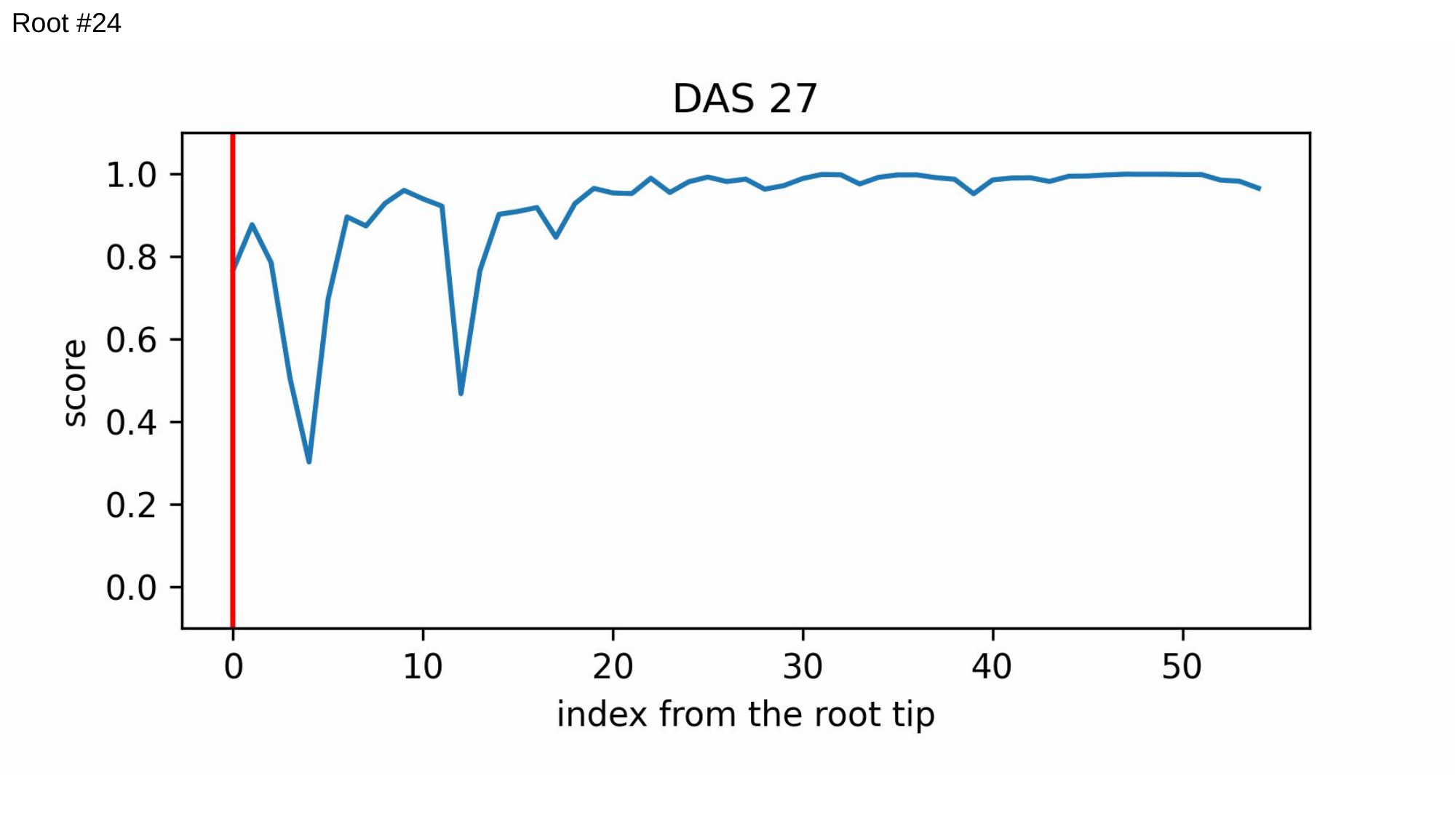

Root #24

## Slide 25
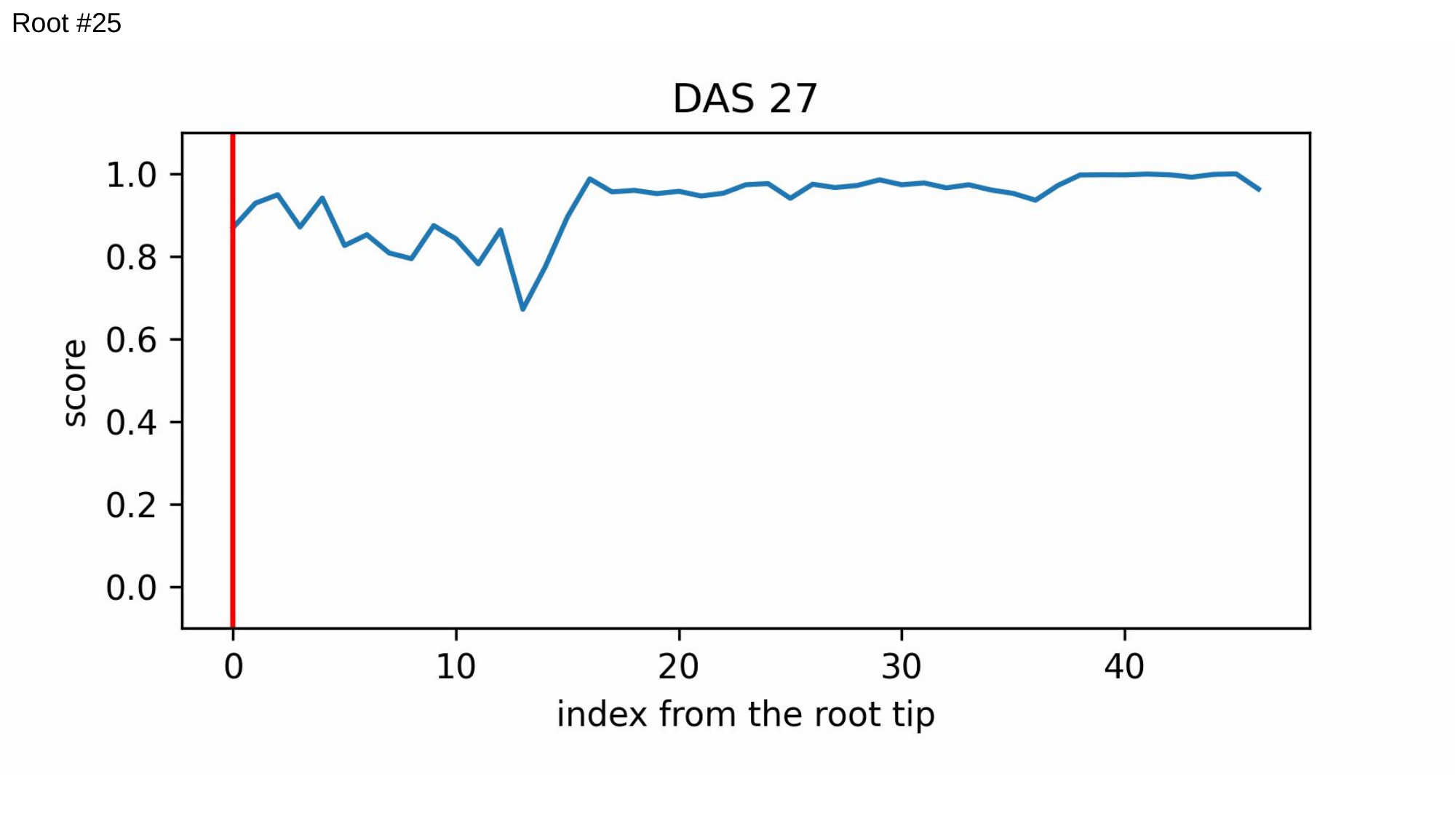

Root #25

## Slide 26
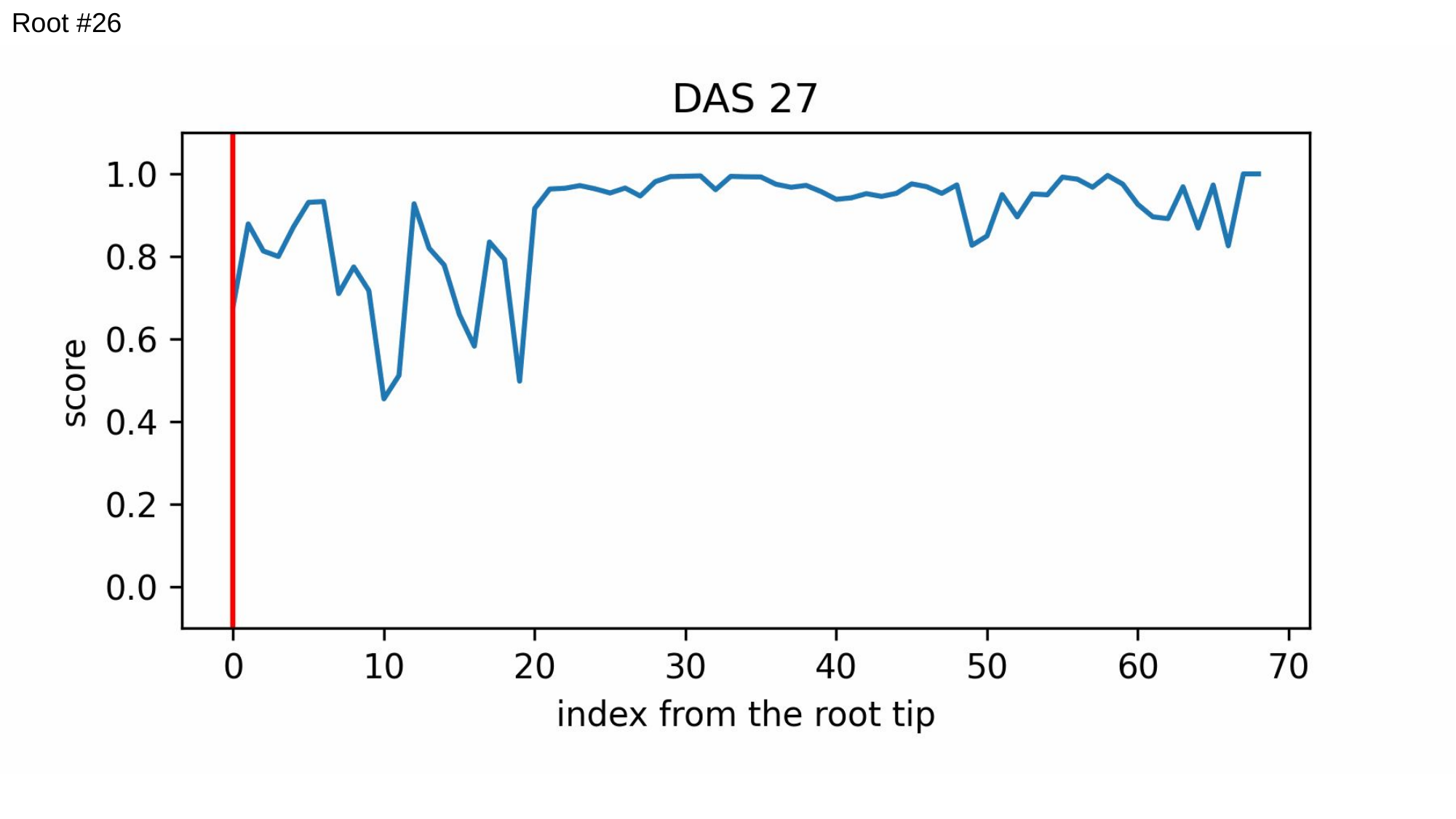

Root #26
